# Inhibition of MurA Enzyme from *Escherichia coli* by Flavonoids and Their Synthetic Analogues

**DOI:** 10.1021/acsomega.3c04813

**Published:** 2023-08-25

**Authors:** Rok Frlan, Martina Hrast, Stanislav Gobec

**Affiliations:** The Department of Pharmaceutical Chemistry, University of Ljubljana, Faculty of Pharmacy, Aškerčeva 7, 1000 Ljubljana, Slovenia

## Abstract

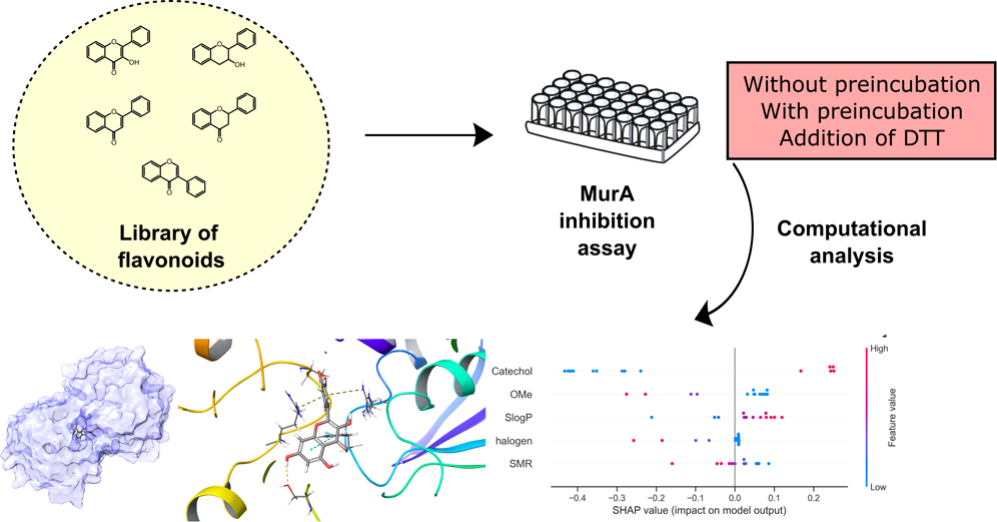

MurA catalyzes the first step of peptidoglycan (PG) biosynthesis and is a validated target for the development of new antimicrobial agents. In this study, a library of 49 plant flavonoids and their synthetic derivatives were evaluated for their inhibitory properties against MurA from*Escherichia coli*. The compounds were tested with and without preincubation and with the addition of DTT to understand the mechanism of inhibition. Thirteen compounds were identified as reversible, time-dependent inhibitors, with inhibition levels ranging from 480 nM to 57 μM, and ampelopsin as the most potent compound. To investigate the major pharmacophore elements responsible for the activity, 2D-QSAR and docking analyzes were performed. The results showed that the catechol moiety and an additional aromatic system were the most important features contributing to the activity of the compounds. However, most of the compounds did not show antibacterial activity against*E. coli* and*Staphylococcus aureus*strains, suggesting that their inhibitory activity against MurA may not be strong enough to induce antibacterial effects. Nevertheless, our results suggest that flavonoids are a promising starting point to develop new inhibitors of MurA and show great potential for further steps in drug development.

## Introduction

Bacterial resistance to antibiotics (AMR) has become a serious public health threat that requires urgent attention.^1^ Although the development of effective antibacterial agents is currently a global priority, the rate of resistance development is outpacing the introduction of new treatments.^2^ One promising strategy to combat AMR is to target underutilized bacterial enzymes involved in essential cellular processes to develop new antimicrobial agents. One such target is the enzyme MurA (UDP-NAG enolpyruvyl transferase), which catalyzes the first step of peptidoglycan biosynthesis, a crucial component of the bacterial cell wall.^3^

MurA is an invaluable target for small-molecule inhibitors because it plays a critical role in bacterial survival and is not found in mammals. Clinical inhibition of MurA currently relies primarily on fosfomycin, a naturally occurring broad-spectrum antibiotic that covalently modifies the enzyme’s catalytic Cys. Although commonly used to treat lower urinary tract infections, the efficacy of fosfomycin has been significantly compromised by the emergence of resistance mechanisms, including overexpression of efflux pumps, altered cell permeability, inactivation by fosfomycin-modifying enzymes, and increased production of UNAG.^4^ Therefore, the urgent development of new inhibitors capable of addressing these challenges is urgently needed.

In the last decade, considerable efforts have been made to identify new MurA inhibitors. Several compounds have been reported, most of which interact with the catalytic Cys via a covalent bond, similar to fosfomycin (for a comprehensive review of covalent MurA inhibitors, see ref (5)). Despite their moderate inhibitory activity in the micromolar range, none of these agents has found its way into the clinic. Against this background, the exploration of natural products for their inhibitory potential has successfully led to the discovery of antibacterial drugs that are still in use today, making them a promising area of research.^6^

Flavonoids, a class of plant secondary metabolites, have attracted considerable attention in recent years because of their impressive range of pharmacological effects. These include potent antibacterial activity against a variety of common Gram-positive and Gram-negative pathogens, including those classified as “ESCAPE bacteria”.^7−9^ In addition, these compounds have demonstrated the ability to enhance bacterial sensitivity to antibiotics and inhibit biofilm formation, making them potential candidates for combination therapies.^10,11^

Flavonoids exert their antibacterial effects through multiple targets, including nucleic acid synthesis, energy metabolism, cell wall synthesis, and the cell membrane.^9^ Their interactions with a variety of targets have raised the question of whether their ontarget activity might result from nonspecific binding rather than binding to the active site.^10,12^ However, studies on nonbacterial enzymes have shown that specificity and selectivity can be achieved with a variety of enzymes.^13−15^ The commercial availability and diverse pharmacological effects of flavonoids have made them attractive for high-throughput and targeted screening campaigns.

**Figure 1:**
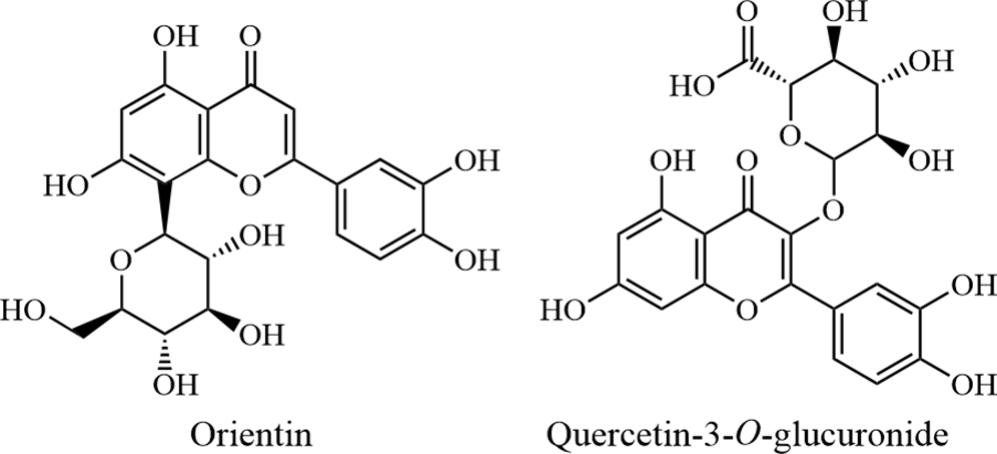
Orientin and quercetin-3-*O*-glucuronide.

Previous reports have identified a limited number of flavonoids that inhibit MurA, including orientin and quercetin-3-*O*-d-glucuronide (Figure 1), which inhibit the enzyme in*Fusobacterium nucleatum* through mixed and uncompetitive modes of inhibition.^16^ In addition, two antibacterial flavonoid compounds have been isolated from Sarang Semut that have also been reported to inhibit MurA.^17^

In this manuscript, we present the results of a screening study of 49 flavonoids against MurA. To the best of our knowledge, this is the first report of screening of a larger library of flavonoids against this enzyme. Our goal was to investigate whether these compounds have potential as novel inhibitors of MurA. In addition, basic pharmacophore elements responsible for their activity were also investigated. The results of our research will provide valuable insights into the potential of flavonoids as inhibitors of bacterial cell wall synthesis.

## Experimental Section

### Chemicals and Reagents

All flavonoids used in this study are presented in Table 1. The compounds were purcased from MolPort, Alfa-Aesar, APIN Chemicals Ltd., Carl Roth, ChromaDex, Fluka, Janssen Chimica, Serva, Sigma-Aldrich, and LGC Standards. To protect them from possible decomposition, they were stored at −18 °C and protected from light. All compounds were dissolved in 100% dimethyl sulfoxide (DMSO) and 10 mM stock solutions were prepared immediately before the biological assay and stored at −20 °C. All other chemicals were ultrapure analytical grade and were purchased from Sigma-Aldrich or Acros Chemical Company.

**Table 1:**
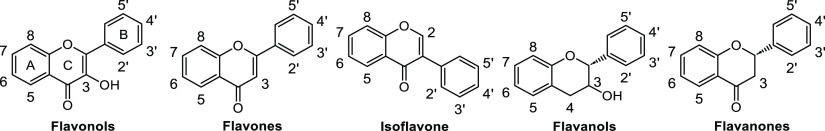
Structures of Flavonoids that Were Used in this Study

	flavonoid	substituents						
no.	flavonols	3	5	7	8	3′	4′	5′
1	rutin	*O*-rutinoside	OH	OH		OH	OH	
2	fisetin			OH		OH	OH	
3	kaempferol		OH	OH			OH	
4	myricetin		OH	OH		OH	OH	OH
5	myricitrin	*O*-rhamnoside	OH	OH		OH	OH	OH
6	hyperoside	*O*-galactoside	OH	OH		OH	OH	
7	isoquercetin	*O*-glucoside	OH	OH		OH	OH	
8	rhamnetin		OH	OCH_3_		OH	OH	
9	quercetin	OH	OH	OH		OH	OH	
10	quercitrin	*O*-rhamnoside	OH	OH		OH	OH	
11	galangin	OH	OH	OH				
12		OCH_3_		OCH_3_		OCH_3_	OCH_3_	
13		OH		OH	CH_3_	OH	OH	
14	kaempferide	OH		OH			OCH_3_	

flavones		5	6	7	8	2′	3′	4′	5′
15		OH		*O*-glucuronide				OH	
16	chrysin	OH		OH			OCH_3_	OH	OCH_3_
17	luteolin	OH		OH			OH	OH	
18		OH		*O*-glucoside			OH	OH	
19			Cl				OCH_3_	OCH_3_	
20			Br				OCH_3_	OCH_3_	
21			CH_3_					Cl	
22			Cl			OCH_3_			
23			Cl					Cl	
24	acacetin	OH		OH				OCH_3_	
25				OH	OH				
26	baicalein	OH	OH	OH					
27	eupatilin	OH	OCH_3_	OH			OCH_3_	OCH_3_	

isoflavone	2	5	7	8	3′	4′
28			OCH_3_			Cl
29	CF_3_	OH	OH			OCH_3_
30		CH_3_	OH			OCH_3_
31			OCH(CH_3_)COOCH_3_			Cl
32	CF_3_		OH			
33			OCH_3_	CH_3_		
34		OH	OH		OH	OCH_3_

flavanols		3	4	5	7	3′	4′
35	leucocyanidin	(*S*)-OH	(*S*)-OH	OH	OH	OH	OH
36	(−)-epicatechin (2R,3R)	(*R*)-OH		OH	OH	OH	OH
37	(+)-catechin	(*S*)-OH		OH	OH	OH	OH

flavanones		3	5	6	7	8	3′	4′	5′
38	hesperetin		OH		OH		OH	OCH_3_	
39	isoxanthohumol		OCH_3_		OH	prenyl		OH	
40	naringin		OH		*O*-rhamnoglucoside			OH	
41	naringenin		OH		OH			OH	
42	bavachinin			prenyl	OCH_3_			OH	
43	bavachin			prenyl	OH			OH	
44			OH		OH	CH_3_			
45	8-prenylnaringenin		OH		OH	prenyl			OH
46	flavanone								
47	taxifolin	OH	OH		OH			OH	OH
48	(−)-alpinetin		OCH_3_		OH				
49	ampelopsin	OH	OH		OH		OH	OH	OH

### Calculation of Physicochemical Properties

We used RDKit nodes implemented in KNIME 4.7.1.18^18^ to calculate the physicochemical properties of our compounds, including SlogP, topological polar surface area (TPSA), molecular weight (MW), number of rotatable bonds (NumRotBonds), number of hydrogen bond donors (NumHBDs), number of hydrogen bond acceptors (NumHBAs), and number of rings (NumRings). Our goal was to investigate whether these descriptors showed differences between our compounds and whether these differences could be related to their activity. In addition, we calculated several other descriptors, including molecular refractive index (SMR), surface area (LabuteASA), number of aromatic rings, number of saturated rings, and number of aliphatic rings, for use in a logistic regression model.

### Calculation of Feature Importances

SMARTS and SMILES functional group definitions were downloaded from GitHub^19^ and combined with functional group definitions from our previous research (Table S4).^20^ The RDKit substructure counter node implemented in KNIME was used to create a 250-bit fingerprint of each flavonoid, with each bit representing several of the corresponding functional groups. The physicochemical features and fingerprints were combined, and compounds that were suspicious in our MurA inhibition assay based on Hill coefficient measurement were removed from the data set. To avoid data leakage, all further data manipulations were performed on the training data set, which was generated by stratifying the data set into training and test data sets in a 7:3 ratio based on activity class. Invariant (standard deviation < 0.01) and highly correlated features (Pearson correlation coefficient > 0.7) were removed, resulting in a data set with 11 features (Table S3). The KBest algorithm was used to assess the significance of the features using the F-test, and the top five features were used to develop a logistic regression model.^21^ To address the imbalance between active and inactive samples in the training data set, the Synthetic Minority Oversampling Technique (SMOTE) was used to increase the number of minority class samples.^22^ We performed hyperparameter optimization with GridSearchCV, using 10-fold cross-validation and the F1-macro metric as the evaluation metric.^23^ The F1-macro metric is useful for imbalanced data sets because it calculates the F1 score for each class and takes the average of these scores, weighting each class equally regardless of the number of instances in the class. The importance of each feature was then calculated using SHapley Additive exPlanations (SHAP) in Python.^24^

### Docking of Quercetin (**9**) to MurA

#### Protein Preparation

We extracted the crystal structure of the dead-end complex of MurA with fosfomycin and UNAG (PDB code3KR6) from the Protein Data Bank and prepared it for analysis with Schrödinger’s Protein Preparation in the Schrödinger Suite.^25^ Preparation included removal of water and co-crystallized molecules such as ligands, automatic assignment of bond orders, addition of hydrogen atoms, conversion of selenomethionines to methionines, addition of missing side chains, creation of disulfide bridges when possible, removal of water beyond a 3 Å radius from heteroatoms and with less than 3 H bonds to non-water, and protonation of heteroatoms at pH 7.0. To optimize the protein for further analysis, we used the impred script to perform constrained minimization with a maximum root-mean-square deviation (RMDS) of 0.30 Å.

#### Preparation of Ligands

We used ChemDraw 18 (PerkinElmer) to draw the modified quercetin (**9**) derivative required for the covalent docking procedure. For this purpose, all three available sites of ring A were monofluorinated. We then converted the three structures to SMILES format using Open Babel.^26^ The resulting structures were imported into the program Maestro^27^ from the Schrödinger Suite. The conformations of the molecules were generated using the OPLS4 force field and ionized using Epik^28^ at a target pH of 7 ± 2. Finally, the conformers were saved in Maestro format for further docking.

#### Docking with CovDock

For docking, we used the CovDock^29^ software from the Schrödinger Suite. We defined Cys115 as a reactive residue and used it as a centroid to define the enclosing box. The size of the box was sufficient to allow docking of ligands up to 30 Å in size. Next, we chose a nucleophilic substitution on a fluorinated ligand as the reaction type. We set a cutoff value of 2.5 kcal/mol energy to maintain docking poses for further refinement with a minimization radius of 3.0 Å. We used MM-GBSA scoring and selected only one pose per ligand reaction site.

### Generation of Figures

The graphical representation of the top-scoring three-dimensional structure of the docking results was created using Maestro software (v. 2021-3). All other plots were created using the Seaborn and Matplotlib libraries in the Jupyter Notebook.

### Statistical Analysis

IC_50_ values were determined by plotting RA values against the applied inhibitor concentration, using GraphPad Prism 7.0 (GraphPad Software, Inc., La Jolla) to fit the experimental data to Hill’s four-parameter dose–response curve. To compare the physicochemical properties of flavonoids, we first tested the normality of the continuous features using D’Agostino’s *K*^2^ test in Python 3.9.12 (Table S1). The results showed that none of the tested features were normally distributed. Therefore, we used the Mann–Whitney U-rank test to compare SlogP, TPSA, AMW, NumRotBonds, NumHBDs, NumHBAs, and NumRings in Python 3.9.12 (Table S2).

### Biochemical Analysis

#### MurA Inhibition Assay

To determine the inhibition of MurA, the colorimetric assay with malachite green was used to measure the release of orthophosphate generated during the reaction.^30^ For the assay, 2.5 μL of each compound tested at a concentration of 2 mM, previously dissolved in DMSO, was added in duplicate to a reaction mixture containing 50 μL of 50 mM HEPES pH 7.8, 0.005% Triton X-114, 200 μM UNAG, 100 μM PEP, and purified MurA (250 nM) diluted in 50 mM HEPES at pH 7.8. Time-dependent inhibition assays were also performed by preincubating MurA with the 200 μM UNAG substrate and target compounds for 30 min at 37 °C before starting the reaction with the addition of the second substrate 100 μM PEP, resulting in a final mixture volume of 50 μL. The reaction was stopped after a 15 min incubation at 37 °C by adding the Biomol reagent (100 μL, Enzo Life Sciences, New York), and the absorbance was measured after 5 min at 650 nm using a microplate reader (BioTek, Synergy H4). The final concentration of DMSO in the reaction mixture was 5% (v/v), and 1 μM fosfomycin was used as a reference in the MurA assay. The percentage of residual activity was calculated in comparison to the negative controls, which contained only 5% DMSO. IC_50_ values were determined by measuring residual activities at 7 different concentrations of the compounds and calculated using GraphPad Prism (GraphPad Software, San Diego, California).

#### MurA Dilution Assay

For the dilution assay, the concentration of compounds tested was 10 times the IC_50_ value (determined after 30 min of preincubation). The enzyme concentration used was the usual concentration in reactivity assays 100-fold (25 μM). After 30 min of preincubation, the mixture was diluted 100-fold, then the previously described reactivity assay was performed using 100 μM PEP.

#### Antibacterial Assays

To test antimicrobial properties, a broth microdilution method in 96-well plates was used according to Clinical and Laboratory Standards Institute guidelines^31^ and European Committee on Antimicrobial Susceptibility Testing (Clinical and Laboratory Standards Institute) recommendations.^32^ Bacterial suspensions, equivalent to a 0.5 McFarland turbidity standard, were diluted with cation-adjusted Mueller–Hinton broth with TES buffering (ThermoFisher Scientific) to achieve a final inoculum of 10^5^ CFU/mL. Compounds dissolved in 100% DMSO at a concentration of 10 mM were mixed with the inoculum and incubated at 35 °C for 20 h. The minimum inhibitory concentrations (MICs) were then determined visually as the lowest dilution of the compounds that showed no turbidity. MICs were determined against two reference bacterial strains, *S. aureus* (ATCC 29213) and *E. coli* (ATCC 25922), using tetracycline as a positive control on each assay plate, with MICs of tetracycline of 0.5 and 1 μg/mL for *S. aureus* and*E. coli*, respectively.

## Results

### Inhibition of MurA by Flavonoids

First, the inhibitory effect of 49 isolated flavonoids, presented in Table 1, on MurA enzyme was investigated. The inhibitory effect was determined using the assay with malachite green, which measures the formation of phosphate in the enzyme reaction. To ensure that the inhibitory effect was specific to the flavonoids and not due to aggregation, a detergent (0.005% Triton X-114) was used in the assay system.^33^

#### First Screening

The initial screening of flavonoids was performed under three different assay conditions: (1) without preincubation, (2) with a 30 min preincubation, and (3) with the addition of dithiothreitol (DTT) along with a 30 min preincubation. These different conditions were necessary to understand the mechanism of inhibition of each flavonoid. By testing the flavonoids with and without preincubation, we were able to determine whether the inhibition was time-dependent, meaning that a stronger binding interaction formed between the enzyme and the inhibitor over time. To distinguish whether the adduct formed between the inhibitor and the enzyme was covalent or noncovalent, the inhibitory activity was tested in the presence of 0.1 mM DTT.

**Figure 2:**
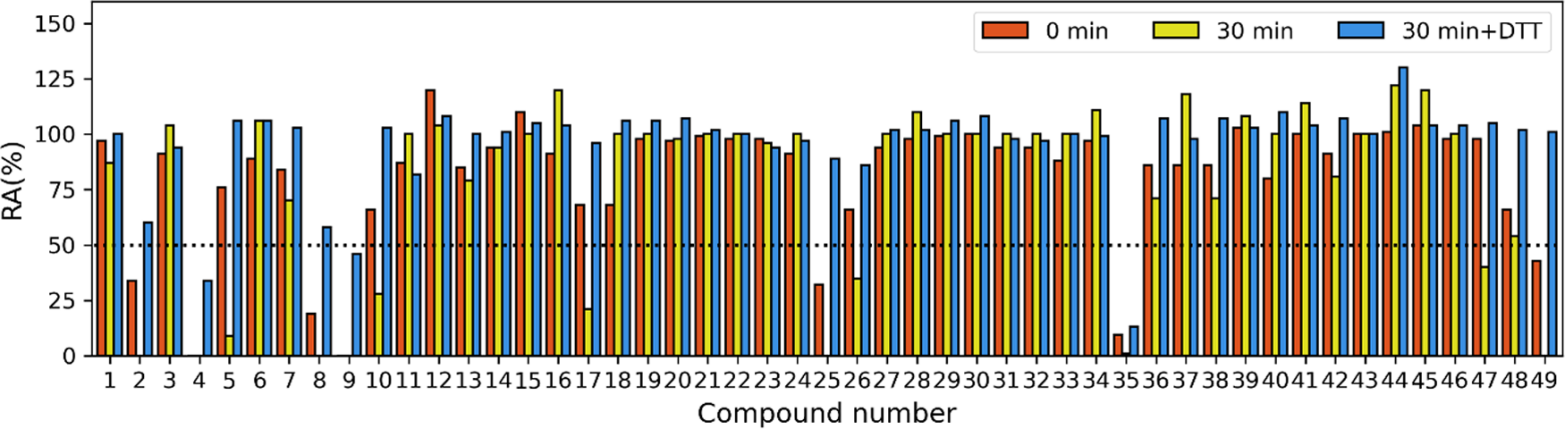
Results of in vitro inhibition of flavonoids on MurA enzyme from *E. coli*. Standard deviation is within ±10% limit. RA: residual activity. Residual activities were determined at 100 μM with (yellow) or without (red) preincubation and with the addition of DTT and 30 min of preincubation (blue). Black dotted line represents an activity treshold of 50% RA.

The results of all three assay conditions are shown in Figure 2 as residual activity at 100 μM for all 49 flavonoids. A threshold of 50% RA (represented by the black dotted line) was used to determine whether a flavonoid was active or inactive. We found that some of the flavonoids, particularly fisetin (**2**), myricetin (**4**), myricitrin (**5**), rhamnetin (**8**), quercetin (**9**), quercitrin (**10**), luteolin (**17**), flavonoid **25**, baicalin (**26**), leucocyanidin (**35**), flavonoid **46**, taxifolin (**47**), and ampelopsin (**49**) showed inhibitory activity on MurA in at least one of the three assays applied. Subsequently, the IC_50_ was determined for all active compounds, and the results are shown in Table 2. The inhibition of MurA by these compounds proved to be time-dependent, as the activity increased after 30 min of preincubation for most compounds that were active in the first assay. In addition, some compounds that were inactive without preincubation (compounds **4**, **10**, **26**, **47**, and **48**) were found to be active after 30 min of preincubation, further demonstrating the time-dependent kinetics of these compounds. The most potent time-dependent inhibitor was the flavonoid ampelopsin (**49**) with an IC_50_ of 480 nM.

**Table 2:** Results of In Vitro Inhibition of Selected Flavonoids on MurA Enzyme from*E. coli*^a^

	RA%^b^ or IC_50_ (Hill coefficient)				MIC (μM)	
cmp.	0 min	30 min	30 min + DTT	dilution assay	*E. coli.*	*S. aureus*
**2**	91 μM (1.4)	8.8 μM (1.0)	60%^c^	98%	>250	>250
**4**	2 μM (0.8)	1.3 μM (1.9)	34%^c^	84%	>250	>250
**5**	76%	2.4 μM (0.6)	106%	97%	>250	>250
**8**	96 μM (1.1)	13 μM (1.1)	58%^c^	77%	>250	>250
**9**	14 μM (0.8)	9.3 μM (1.3)	46%^c^	93%	>250	>250
**10**	66%	33 μM (1.1)	103%	88%	>250	>250
**17**	68%^c^	41.5 μM (1.3)	96%	91%	>250	125
**25**	88 μM (2)	12.9 μM (1.4)	89%	100%	>250	125
**26**	66%	61 μM (1.4)	86%	50%	>250	31
**35**	69 μM (2.6)	28.6 μM (2.9)	13%^c^	ND^c^	>250	>250
**47**	98%	4.5 μM (0.5)	105%	93%	>250	>250
**48**	66%^c^	57 μM (1.6)	102%	93%	>250	>250
**49**	36 μM (0.8)	0.48 μM (0.8)	101%	100%	>250	125

a Standard deviation is within ±10% limit.

b RA: residual activity. Residual activities were determined at 100 μM without (0 min) preincubation, with 30 min preincubation, with addition of DTT and 30 min preincubation, and in dilution test after 30 min.

c High background or poor solubility. ND: not determined.

#### Hill Coefficient

**Figure 3:**
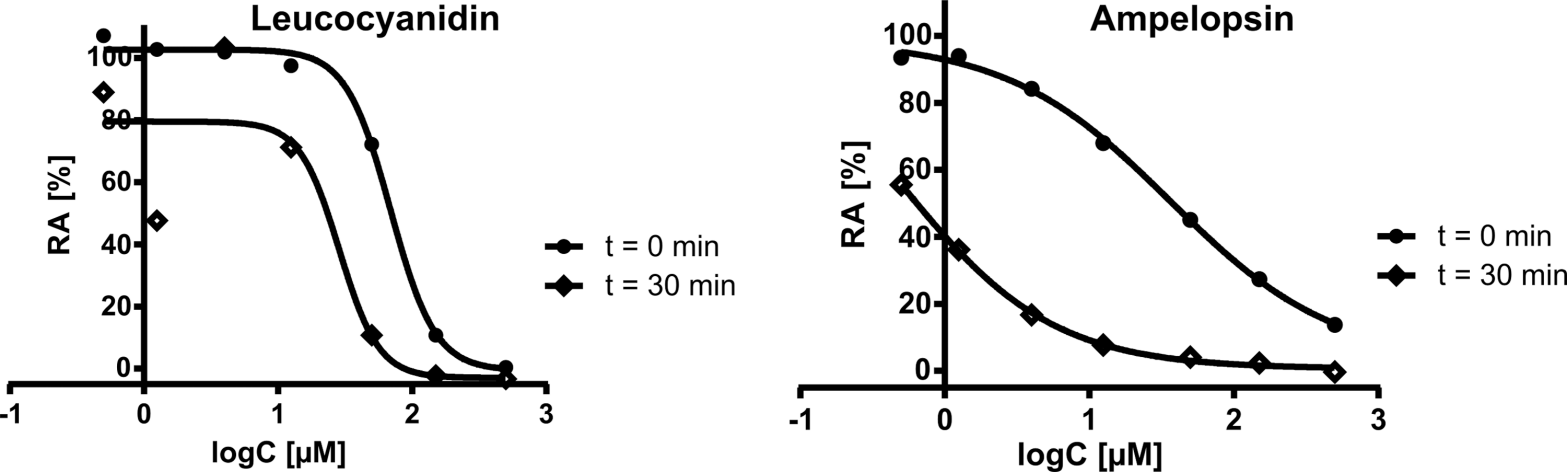
Dose–response curves of leucocyanidin (**35**) and ampelopsin (**49**) measured at 0 and 30 min.

To evaluate possible nonspecific protein interactions between the flavonoids and the enzyme, we calculated the Hill coefficient from the dose–response curves (Table 2 and Figure S29). A Hill coefficient greater than 1 indicates that inhibition of the enzyme is cooperative, that is, the presence of one bound molecule increases the likelihood that others will also bind to the enzyme. Such interactions may reflect nonspecific binding of the activating compound to the enzyme and lead to false-positive results.^34^ A Hill coefficient greater than 1 was observed for some compounds, indicating a potential for nonspecific interactions. Considering the Hill coefficient, we found that the activities of some compounds, including myricetin (**4**), flavonoid **25**, leucocyanidin (**35**), and taxifolin (**47**), are suspicious and should be interpreted with great caution because their activity may be due only to denaturation of the enzyme. Figure 3 shows the difference between the dose–response curves after 0 and 30 min of leucocyanidin (**35**) with a very high Hill coefficient (>2.5) and ampelopsin (**49**) with a Hill coefficient close to 1. For leucocyanidin, a significant decrease of RA is observed, whereas ampelopsin shows a gradual decrease of RA with decreasing concentration.

#### Mechanism of Action

To investigate whether the inhibition of MurA enzyme by flavonoids was due to covalent or tight-binding interactions, we added DTT to the assays. DTT stabilizes the enzyme, acts as a radical scavenger, reacts with electrophilic compounds, and can also reduce covalent bonds. Specifically, if the inhibitory activity was based on a covalent interaction, the compounds’ warheads would react with Cys115, but this interaction could be reversed by DTT.^35^ Our results demonstrated that the inhibitory activity of most tested flavonoids was significantly reduced in the presence of DTT, indicating that their interaction with MurA was predominantly covalent. While we could not reliably measure the IC_50_ of fisetin (**2**), myricetin (**4**), rhamnetin (**8**), quercetin (**9**), and leucocyanidin (**35**) because of low solubility or high background, it was possible to compare the RAs, which also showed reduced inhibition in the presence of DTT.

To determine the extent of covalent interaction with the enzyme and to better understand its nature, we performed a dilution assay with the tested compounds. The results showed that flavonoid activity decreased with dilution for most compounds, implying a reversible nature of the covalent inhibition. Moreover, the activity was completely abolished for most compounds. However, baicalein (**26**) retained some inhibitory activity, suggesting that the covalent adduct formed between this compound and the enzyme is more stable than the complexes formed between the enzyme and other flavonoids.

### Physicochemical Properties

**Figure 4:**
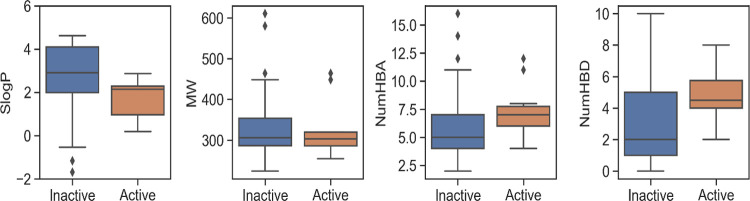
Comparison of differences between active (orange) and inactive (blue) compounds with respect to the four rule-of-5 descriptors. A statistically significant difference was found between the two classes for the number of hydrogen bond acceptors (NumHBAs), TPSA, and log *P*. However, no differences were found between the two classes for the number of hydrogen bond donors (NumHBDs).

To compare active and inactive flavonoids, we evaluated various physicochemical properties. We performed statistical analysis using the Mann–Whitney U-rank test (Table S2) and also generated graphical representations of the distribution of four representative basic properties (Figure 4). Our analysis revealed significant differences between the two groups for TPSA, log *P*, NumRotBonds, and NumHBDs (*p* < 0.05). In particular, the active compounds were more polar and had a higher TPSA value, lower log *P* value, and lower NumRotBond value. However, no significant differences were found in MW, NumberHBAs, or the number of rings between the active and inactive compounds.

### Structural Properties and Feature Importances

#### KBest

**Figure 5:**
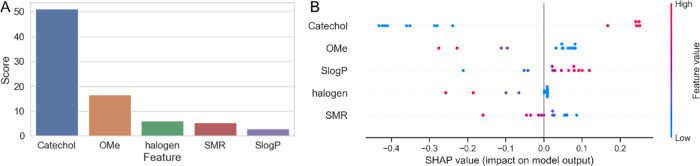
(A) Results of KBest feature selection algorithm. (B) Summary plot of SHAP values for five most important features.

To better understand which functional groups are important for activity, we created a data set of flavonoids with the corresponding definitions of functional groups and physicochemical properties. After cleaning, the data set contained a total of 11 features.^36,37^ We used KBest in Python to rank the features based on the *F*-test between the feature and the activity variable,^23^ and found that the presence of catechol functions was most important for activity.^38^ Other features that were significantly less important were the presence of methoxy, halogen, and allyl oxide groups and log *P* value (Figure 5a).^39^

#### SHAP

While KBest is a useful method for selecting the best *k* features based on a statistical test, it does not provide information on how each feature contributes to the outcome of the model for specific instances. Therefore, in this study, we selected the five best features from the KBest ranking and built a QSAR classification model with logistic regression (LR). We optimized the hyperparameters of the model using GridSearch and the model achieved an average F1-macro score of 0.851 ± 0.189 and 0.789 on the train and test sets, respectively. Next, we used SHAP to visualize feature importances.^24^ SHAP is a method that provides individual feature importance scores, taking into account interactions between features. It is based on the concept of Shapley values from cooperative game theory. The results of the SHAP analysis are shown in Figure 5b, where the *x*-axis represents the SHAP value and the *y*-axis represents the selected features. Each dot in the graph represents a SHAP value for a predictive feature, with red indicating a higher value of a feature and blue indicating a lower value. The distribution of red and blue dots gives us an idea of the overall impact of each feature on the model’s outcome. Our results show that increasing the number of catechol groups increases the probability of activity in a LR model, whereas the opposite is true for decreasing the number of catechol groups. Interestingly, using SHAP, we observed that higher lipophilicity of compounds increases the probability that a compound is active, although it was previously shown that active compounds have lower log *P*. This discrepancy could be a result of different approaches and assumptions used in both methods. For example, SHAP estimates the contribution of each feature to the output of the LR model, whereas the statistical analysis only compares the mean values of log *P* between active and inactive compounds. Additionally, there may be nonlinear relationships between the features and the activity of compounds, which could also lead to different results. We also found that the presence of three functional groups (halogen, allyl oxide, and OMe) has a negative effect on activity.

### Covalent Docking of Quercetin (**9**) to MurA

To structurally explain the binding of flavonoids to the MurA enzyme, a computational analysis of quercetin (**9**) was performed using the crystal structure of MurA in a complex with the irreversible inhibitor fosfomycin (PDB: 3KR6) and UNAG. CovDock from Schrödinger was used for ligand preparation and docking analysis.^29^

**Figure 6:**
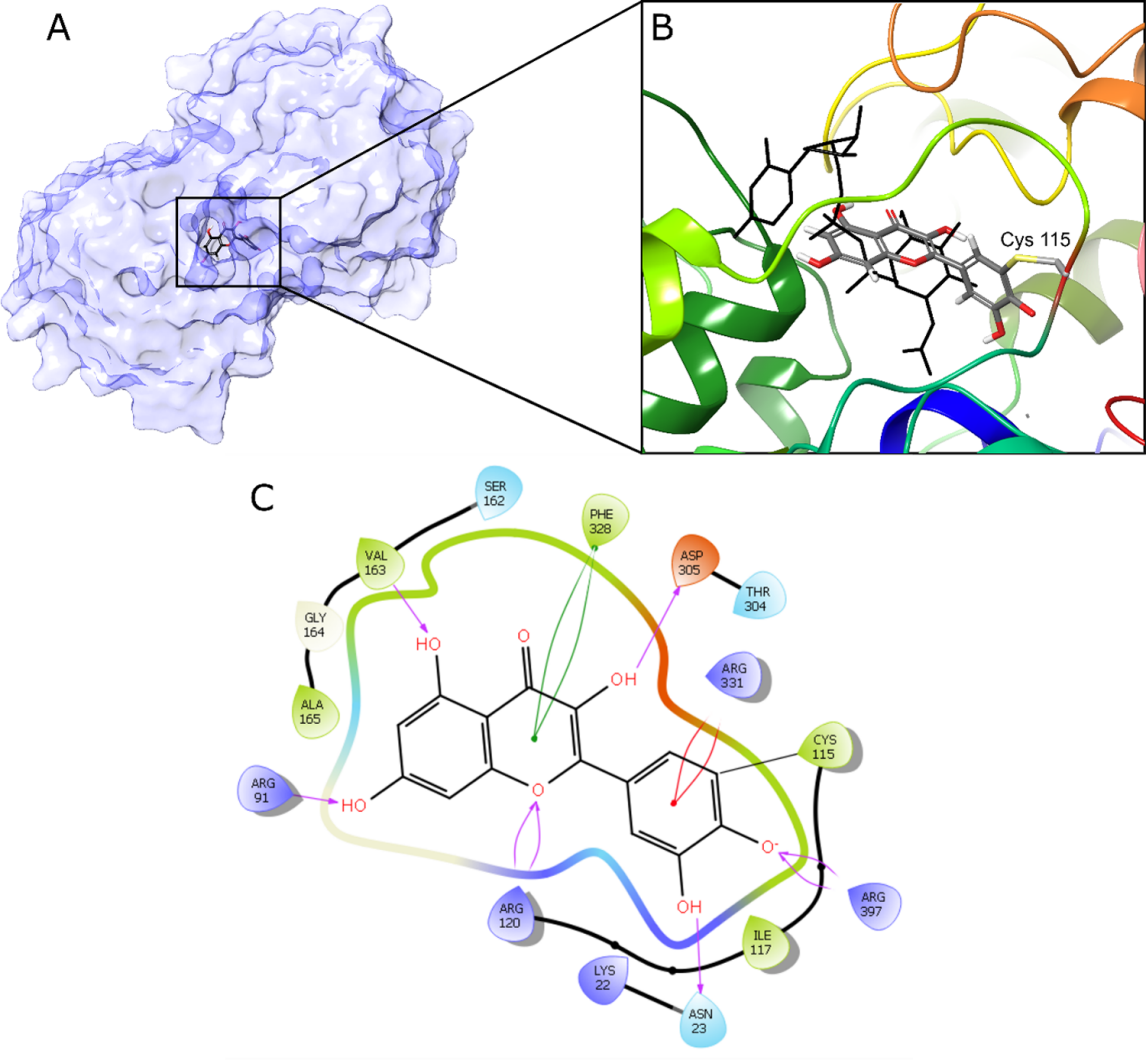
Binding mode of quercetin (**9**). (A) Protein surface with quercetin bound to UNAG and PEP binding site. (B) Close-up view of quercetin in the binding pocket in comparison with the UNAG binding mode (black line presentation). (C) Ligand interaction diagram. Interactions with the surrounding amino acids are presented as colored arrows.

Figure 6 presents the 2D and 3D representations of the highest-scoring binding mode of quercetin (**9**) as revealed by the docking analysis of MurA. The 3D representation shown in panels A and B depicts the protein surface and inhibitor in a closed conformation of MurA and an enlarged view of the quercetin binding site compared with that of the native ligand UNAG, respectively. The rod representation of Cys115, which covalently links to the catechol ring (ring B) of quercetin, is also shown in panel B, alongside the protein structure as a colored ribbon diagram. The 2D interaction diagram in panel C provides a detailed view of the interactions between quercetin and the surrounding amino acids.

Analysis of the binding mode indicates that quercetin (**9**) adopts a conformation in which its catechol moiety (ring B) is directed toward the exterior of the enzyme, whereas its rings A and C are directed toward the binding pocket for the uridine portion of UNAG. Quercetin (**9**) forms several hydrogen bonds and hydrophobic interactions with the surrounding residues. Ring B, covalently bonded to Cys115, forms a π-cation interaction with Arg331 and hydrogen bonds between the hydroxyl groups on the ring and Arg397 and Asn23, respectively. In addition, both rings A and C form a T-shaped π–π interaction with Phe328 and hydrogen bonds between the three hydroxyl groups on both rings with Arg91 and Val163, both of which form the diphosphate-binding pocket, and Asp305, which is in the glucosamine-binding pocket. Furthermore, a hydrogen bond is formed between Arg120 and the cyclic oxygen of ring C.

### Antibacterial Assays

All selected flavonoids were tested for their ability to inhibit the growth of*E. coli* and*S. aureus* using the broth microdilution method, and the results are shown in Table 2. Of all the flavonoids tested, only baicalein (**26**) inhibited *S. aureus* with an MIC of 31 μM and had no effect on*E. coli* growth. All other flavonoids had very low inhibitory potential or did not inhibit the growth of either bacteria at all.

## Discussion

The emergence of drug-resistant bacteria has greatly reduced the efficacy of current antibiotics, resulting in increased morbidity, mortality, and healthcare costs. To address this challenge, underexploited targets such as MurA have been identified as promising alternatives to novel antibacterial methods, such as the CRISPR-Cas system.^40^ Covalent inhibition of MurA, for example, with the clinically used fosfomycin, is a validated strategy for antibacterial drug development. However, despite numerous reports of covalent inhibitors, no other MurA inhibitor has found its way into the clinic. Therefore, the search for more effective MurA inhibitors is both urgent and highly desirable, as they could provide an effective solution to combat drug-resistant bacterial infections, potentially saving countless lives and reducing the burden on healthcare systems.

In this manuscript, a screening of 49 plant flavonoids and their semisynthetic derivatives was performed. The inhibitory activity of these flavonoids was tested under three different conditions necessary to understand the mechanism of inhibition of each flavonoid. Our results suggest that flavonoids are promising as potential inhibitors of the target enzyme because, of all the compounds tested, 13 were identified as time-dependent inhibitors of MurA, with inhibitory potency ranging from 480 nM to 57 μM. Thus, the flavonoid inhibitors identified in this study show high potential for further investigation in drug design. However, nonspecific protein interactions could be a potential limitation in the interpretation of the results.

We used the Hill coefficient to investigate the probability of nonspecific interactions between flavonoids and the enzyme. The Hill coefficient is commonly used to determine the degree of cooperativity in a biological system, which may indicate the presence of nonspecific interactions with the enzyme. Previous studies have reported a phenomenon in which binding of multiple flavonoid molecules to different sites on the enzyme results in nonspecific denaturation of the enzyme.^34,41^ This process has been documented and is used in the tanning of animal skins for the production of leather, where flavonoids react nonspecifically with biological macromolecules.^34^ We observed a Hill coefficient greater than 1 for several flavonoids, including myricetin (**4**), flavonoid **25**, leucocyanidin (**35**), and (−)-alpinetin (**48**), indicating the potential for nonspecific binding. These results suggest that these compounds may have induced denaturation of the enzyme, leading to false-positive results. Nevertheless, the Hill coefficient has its limitations, and its interpretation should be taken with caution because a Hill coefficient greater than 1 only indicates that the presence a molecule bound to an enzyme increases the likelihood that other molecules will also bind to the same enzyme. Therefore, additional studies, such as X-ray crystallography, are needed to confirm that the inhibition is not due to denaturation of the enzyme.

The addition of DTT can stabilize the protein and react with reactive molecules, and this has a significant impact on the inhibitory effect of most flavonoids tested. These results indicate that the warheads of these compounds react primarily with Cys115, suggesting that the inhibition of MurA by flavonoids is due to covalent interactions.

To gain further insight into the covalent interaction between flavonoids and MurA, a dilution assay was performed. The results revealed that the activity of most flavonoids tested decreased upon dilution, indicating that the covalent inhibition was reversible. However, baicalein (**26**) was the only compound that retained some inhibitory activity, suggesting that the covalent interaction is not difficult to reverse for these compounds. Overall, these findings highlight the importance of understanding how flavonoids interact with enzymes and the impact this may have on the effectiveness of these compounds as inhibitors.

To identify the major pharmacophore elements involved in the inhibition of MurA, we performed 2D-QSAR and docking analysis. However, to ensure the validity of our results, we excluded compounds with a Hill coefficient greater than 1.5 to avoid erroneous conclusions due to potentially false-positive results. Our QSAR study identified a functional group responsible for covalent inhibition of MurA, and both the SHAP analysis and the *F*-test confirmed that the catechol moiety was the most important feature contributing to the activity of the compounds.

At first glance, it may seem unclear how a catechol can act as a warhead, but this moiety can be oxidized to a reactive orthoquinone, which can then form a covalent adduct with Cys115. This idea is supported not only by theoretical considerations but also by recent experimental results in the development of covalent SARS-CoV-2 inhibitors, where catechol has been identified as the key functional group responsible for the activity.^35^

Previous studies have highlighted the importance of the number and substitution pattern of hydroxyl groups in flavonoids for their inhibitory properties against other enzymes.^15,42^ Our observations support this notion, as our QSAR study suggests that the presence of at least two adjacent hydroxyl groups on one of the aromatic rings is a crucial pharmacophore for covalent binding to MurA. In addition, some studies have focused on the geometry and nature of the flavonoid skeleton, but we did not detect a clear pattern suggesting that the flatness or size of the flavonoid skeleton has a significant effect on the inhibition of MurA.^43,44^

However, QSAR analysis can only determine how small changes in 1D and 2D molecular descriptors affect activity. To gain a better understanding of the behavior of these compounds in the 3D chemical space, we performed docking studies focused specifically on quercetin (**9**) to investigate its interactions with the active site of MurA. Our results show that the hydroxyl groups of the flavonoid system are crucial for the formation of hydrogen bonds with the surrounding amino acids, while the aromatic rings of the flavonoid system contribute to the formation of π-cation and T-shaped π–π interactions with the surrounding amino acids. This information could not be obtained from the QSAR study because the flavonoid ring system was held constant for all compounds.

In summary, both our QSAR and docking studies consistently highlight a crucial pharmacophore pattern consisting of a catechol ring and additional aromatic rings. This pharmacophore pattern is critical for the covalent binding of flavonoids to MurA and provides important insights into the mechanism of inhibition of MurA by flavonoids.

In the last part of our study, we investigated the antibacterial activity of the compounds against*E. coli* and*S. aureus*. Many flavonoids have been previously characterized for their antibacterial activities against a variety of common Gram-positive and Gram-negative pathogens in vitro.^7,45^ However, our results showed a lack of activity against *E. coli* and *S. aureus*, suggesting that their inhibitory activity against MurA may not be strong enough to cause antibacterial effects. In particular, flavonoid **25**, luteolin (ref (46), 50 μg/mL) (**17**), baicalein (**26**), and ampelopsin (**49**) showed weak inhibition of *S. aureus* growth, with baicalein (**26**) being the most potent with an MIC of 31 μM. These results suggest that the inhibitory effect of some of these compounds against MurA may contribute to their antibacterial activity, but further studies are needed to substantiate this claim. We acknowledge that there is a discrepancy between our results and previous reports on the antibacterial activity of myricetin (**4**) and quercitrin (**10**) and luteolin (**17**) against *S. aureus*.^46−48^ However, these differences could be due to variations in bacterial strains and assay methods used in the different studies.^49^ Furthermore, the observed discrepancy between the in vitro enzymatic assay and antibacterial activity is not uncommon and could be due to intrinsic bacterial resistance mechanisms such as active antimicrobial efflux, decreased penetration of the drug into cells, or enzymatic metabolism of the antimicrobial agents.^50^

Our results highlight the complexity of structure–activity relationships in flavonoids and underscore the need for more comprehensive research into the structural features that contribute to the inhibitory activity against MurA. The results of our research provide a starting point for the development of new lead compounds with antibacterial activity. Future research should focus on modifying the flavonoid ring system and optimizing potency as a first step. Additionally, a broader range of flavonoid derivatives should be investigated, considering various structural modifications, such as bioisosteric replacement of the flavonoid ring system, which could lead to compounds with improved selectivity, potency, and safety profiles. The derivation or synthesis of closely related structural analogues is a strategy that has already been successfully used in the preparation of (+)-catechin^51^ and myricetin^52^ derivatives with enhanced antibacterial activity. Furthermore, bioisosteric replacement of the flavonoid ring system could further lead to compounds with improved selectivity, potency, and safety profiles.

## Conclusions

In conclusion, we present the results of screening 49 flavonoids of natural origin and their synthetic analogues with respect to inhibition of the bacterial enzyme MurA, which is involved in peptidoglycan biosynthesis. Our study identified 13 flavonoids as potential reversible covalent inhibitors of MurA. We tested these inhibitors under three different conditions to understand the inhibitory mechanism of each flavonoid. Their inhibitory potencies (IC_50_ values) ranged from 480 nM to 57 μM. Our further computational analysis using SHAP and docking studies identified the catechol moiety and an additional aromatic ring as important pharmacophoric elements for activity. Although most flavonoids did not exhibit antibacterial activity against *E. coli* and *S. aureus*, our study highlights the potential of flavonoids as a source of novel starting points in development of antimicrobial agents. This provides a basis for further development and optimization of structurally novel antimicrobial agents to combat increasing bacterial resistance.

## Supporting Information Available

Data analysis and comparison of active and inactive compounds: evaluation of feature distribution and normality (results of D’Agostino’s *K*^2^ test for data normality and Mann–Whitney *U* test results for active and inactive compounds); feature selection: KBest algorithm and *F*-test (features selected using KBest algorithm and *F*-test), ROC–AUC plot of a logistic regression model (ROC–AUC curve of a logistic regression model), and definitions of functional groups; compound characterization: general section, analytical data and chromatograms (^1^H-NMR spectrum of fisetin (**2**), ^13^C-NMR spectrum of fisetin (**2**), HPLC chromatogram of fisetin (**2**), ^1^H-NMR spectrum of myricetin (**4**), ^13^C-NMR spectrum of myricetin (**4**), HPLC chromatogram of myricetin (**4**), ^1^H-NMR spectrum of quercetin (**9**), ^13^C-NMR spectrum of quercetin (**9**), HPLC chromatogram of quercetin (**9**), ^1^H-NMR spectrum of quercitrin (**10**), ^13^C-NMR spectrum of quercitrin (**10**), HPLC chromatogram of quercitrin (**10**), ^1^H-NMR spectrum of luteolin (**17**), ^13^C-NMR spectrum of luteolin (**17**), HPLC chromatogram of luteolin (**17**), ^1^H-NMR spectrum of 7,8-dihydroxyflavone (**25**), ^13^C-NMR spectrum of 7,8-dihydroxyflavone (**25**), HPLC chromatogram of 7,8-dihydroxyflavone (**25**), ^1^H-NMR spectrum of baicalein (**26**), ^13^C-NMR spectrum of baicalein (**26**), HPLC chromatogram of baicalein (**26**), ^1^H-NMR spectrum of alpinetin (**48**), ^13^C-NMR spectrum of alpinetin (**48**), HPLC chromatogram of alpinetin (**48**), ^1^H-NMR spectrum of ampelopsin (**49**), ^13^C-NMR spectrum of ampelopsin (**49**), and HPLC chromatogram of ampelopsin (**49**)); and biochemical analysis: dose–response curves (PDF)
