# Supplementary material for: Inhibition of MurA Enzyme from Escherichia coli by Flavonoids and Their Synthetic Analogues
Source: ACS Omega. 2023 Aug 25;8(36):33006–16. doi: 10.1021/acsomega.3c04813 (PMC10500568; doi:10.1021/acsomega.3c04813)
Supplement: Supplementary file 1 — ao3c04813_si_001.pdf [file ao3c04813_si_001.pdf]

Supporting information

# Inhibition of MurA Enzyme from *Escherichia coli* by Flavonoids and their Synthetic Analogs

*Rok Frlan,* Martina Hrast and Stanislav Gobec**

*University of Ljubljana, Faculty of Pharmacy, Department of Pharmaceutical Chemistry, Aškerčeva 7, 1000 Ljubljana, Slovenia*

# Contents

Data Analysis and Comparison of Active and Inactive Compounds ....... 2
- Evaluation of Feature Distribution and Normality ....... 2
- Comparison of Active and Inactive Compounds ....... 2

Feature Selection ....... 2
- KBest Algorithm and F-test ....... 2
- ROC-AUC plot of a logistic regression model ....... 3
- Definitions of functional groups ....... 3

Compound characterization ....... 9
- General section ....... 9
- Analytical data and chromatograms ....... 10

Biochemical analysis ....... 29

# Data Analysis and Comparison of Active and Inactive Compounds

## Evaluation of Feature Distribution and Normality

**Table S1.** Results of D'Agostino's $K^2$ test for Data Normality

| | Active compounds | | Inactive compounds | |
|---|---|---|---|---|
| | **K-squared value** | **p value** | **K-squared value** | **p value** |
| SlogP | 1.892 | 0.389 | 7.247 | 0.027 |
| TPSA | 1.334 | 0.513 | 10.255 | 0.006 |
| MW | 4.967 | 0.083 | 24.051 | 0.000 |

## Comparison of Active and Inactive Compounds

**Table S2.** Mann-Whitney U test results for Active and Inactive Compounds

| | **U statistic** | **p value** |
|---|---|---|
| SlogP | 100 | 0.019 |
| TPSA | 290 | 0.020 |
| AMW | 171 | 0.560 |
| NumRotBonds | 111 | 0.030 |
| NumHBD | 286 | 0.024 |
| NumHBA | 268 | 0.071 |
| NumRings | 202 | 0.802 |

# Feature Selection

## KBest Algorithm and F-test

**Table S3.** Features selected using KBest Algorithm and F-test

| **Features** | **Score** | **p-value** |
|---|---|---|
| Catechol | 51.558 | 8.410 |
| OMe | 9.686 | 2.505 |
| Halogen | 6.000 | 1.745 |
| SlogP | 4.077 | 1.309 |
| All oxide | 2.087 | 0.810 |
| ArMe | 2.087 | 0.810 |
| NArRings | 1.932 | 0.767 |
| SMR | 1.590 | 0.671 |
| Bromine | 1.000 | 0.492 |
| p-OH | 1.000 | 0.492 |
| -C(=O)O | 1.000 | 0.492 |

## ROC-AUC plot of a logistic regression model

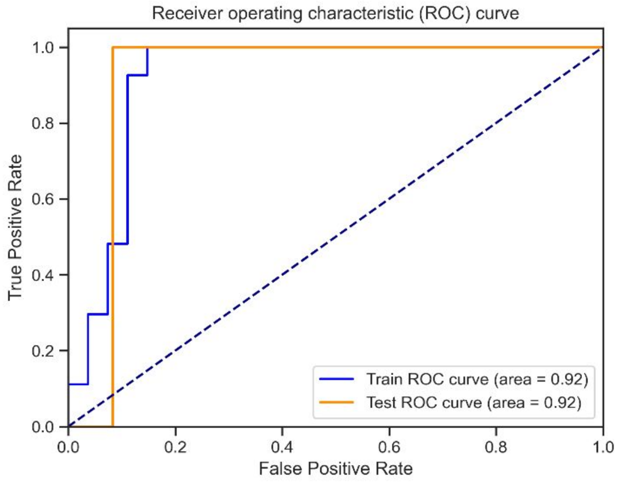


**Figure S1.** ROC-AUC curve of a logistic regression model

## Definitions of functional groups

**Table S4.** Smarts definitions of functional groups

| **functional group** | **smarts** |
|---|---|
| C=O | [CX3]=[OX1] |
| C=O_noCOO | [C!$(C-[OH])]=O |
| Al_OH | [C!$(C=O)]-[OH] |
| Ar_OH | c[OH1] |
| methoxy | [OX2](-[#6])-[CH3] |
| oxime | [CX3]=[NX2]-[OX2] |
| ester | [#6][CX3](=O)[OX2H0][#6] |
| Al_COO | C-C(=O)[O;H1,-] |
| Ar_COO | c-C(=O)[O;H1,-] |
| COO | [#6]C(=O)[O;H,-1] |
| COO2 | [CX3](=O)[OX1H0-,OX2H1] |
| ketone | [#6][CX3](=O)[#6] |
| ether | [OD2]([#6])[#6] |
| phenol | [OX2H]-c1ccccc1 |
| aldehyde | [CX3H1](=O)[#6] |
| quatN | [$([NX4+]),$([NX4]=*)] |
| NH2 | [NH2,nH2] |
| NH1 | [NH1,nH1] |
| NH0 | [NH0,nH0] |
| Ar_N | n |
| Ar_NH | [nH] |
| aniline | c-[NX3;!$(N=*)] |
| Imine | [Nv3](=C)-[#6] |
| nitrile | [NX1]#[CX2] |
| hydrazine | [NX3]-[NX3] |

| | |
|---|---|
| hydrazone | C=N-[NX3] |
| nitroso | [N!$(N-O)]=O |
| N-O | [N!$(N=O)](-O)-C |
| nitro | [$([NX3](=O)=O),$([NX3+](=O)[O-])][!#8] |
| azo | [#6]-N=N-[#6] |
| diazo | [N+]#N |
| azide | [$(*-[NX2-]-[NX2+]#[NX1]),$(*-[NX2]=[NX2+]=[NX1-])] |
| amide | C(=O)-N |
| priamide | C(=O)-[NH2] |
| amidine | C(=N)(-N)-[!#7] |
| guanido | C(=N)(N)N |
| imide | N(-C(=O))-C=O |
| isocyan | N=C=O |
| isothiocyan | N=C=S |
| thiocyan | S-C#N |
| halogen | [#9,#17,#35,#53] |
| alkyl_halide | [CX4]-[Cl,Br,I,F] |
| sulfide | [SX2](-[#6])-C |
| SH | [SH] |
| C=S | C=[SX1] |
| sulfone | S(=,-[OX1;+0,-1])(=,-[OX1;+0,-1])(-[#6])-[#6] |
| sulfonamd | N-S(=,-[OX1;+0,-1])(=,-[OX1;+0,-1])-[#6] |
| prisulfonamd | [NH2]-S(=,-[OX1;+0;-1])(=,-[OX1;+0;-1])-[#6] |
| barbitur | C1C(=O)NC(=O)NC1=O |
| urea | C(=O)(-N)-N |
| term_acetylene | C#[CH] |
| imidazole | n1cncc1 |
| furan | o1cccc1 |
| thiophene | s1cccc1 |
| thiazole | c1scnc1 |
| oxazole | c1ocnc1 |
| pyridine | n1ccccc1 |
| piperdine | N1CCCCC1 |
| piperzine | N1CCNCC1 |
| morpholine | O1CCNCC1 |
| lactam | N1C(=O)CC1 |
| lactone | [C&R1](=O)[O&R1][C&R1] |
| tetrazole | c1nnnn1 |
| epoxide | O1CC1 |
| unbrch_alkane | [R0;D2][R0;D2][R0;D2][R0;D2] |
| bicyclic | [R2][R2] |
| benzene | c1ccccc1 |
| phos_acid | [$(P(=[OX1])([$([OX2H]),$([OX1-]),$([OX2]P)])([$([OX2H]),$([OX1-]),$([OX2]P)])[$([OX2H]),$([OX1-]),$([OX2]P)]),$([P+]([OX1-])([$([OX2H]),$([OX1- |

| | |
|---|---|
| | ]),$([OX2]P)])([$([OX2H]),$([OX1-]),$([OX2]P)])[$([OX2H]),$([OX1-]),$([OX2]P)])] |
| phos_ester | [$(P(=[OX1])([OX2][#6])([$([OX2H]),$([OX1-]),$([OX2][#6])])[$([OX2H]),$([OX1-]),$([OX2][#6]),$([OX2]P)]),$([P+]([OX1-])([OX2][#6])([$([OX2H]),$([OX1-]),$([OX2][#6])])[$([OX2H]),$([OX1-]),$([OX2][#6]),$([OX2]P)])] |
| nitro_arom | [$(c1(-[$([NX3](=O)=O),$([NX3+](=O)[O-])])ccccc1)] |
| nitro_arom_nonortho | [$(c1(-[$([NX3](=O)=O),$([NX3+](=O)[O-])])ccccc1);!$(cc-!:*)] |
| dihydropyridine | [$([NX3H1]1-C=C-C-C=C1),$([Nv3]1=C-C-C=C-C1),$([Nv3]1=C-C=C-C-C1),$([NX3H1]1-C-C=C-C=C1)] |
| phenol_noOrthoHbond | [$(c1(-[OX2H])ccccc1);!$(cc-!:[CH2]-[OX2H]);!$(cc-!:C(=O)[O;H1,-]);!$(cc-!:C(=O)-[NH2])] |
| Al_OH_noTert | [$(C-[OX2H]);!$([CX3](-[OX2H])=[OX1]);!$([CD4]-[OX2H])] |
| benzodiazepine | [c&R2]12[c&R1][c&R1][c&R1][c&R1][c&R2]1[N&R1][C&R1][C&R1][N&R1]=[C&R1]2 |
| para_hydroxylation | [$([cH]1[cH]cc(c[cH]1)~[$([#8,$([#8]~[H,c,C])])]),$([cH]1[cH]cc(c[cH]1)~[$([#7X3,$([#7](~[H,c,C])~[H,c,C])])]),$([cH]1[cH]cc(c[cH]1)-!:[$([NX3H,$(NC(=O)[H,c,C])])])] |
| allylic_oxid | [$(C=C-C);!$(C=C-C-[N,O,S]);!$(C=C-C-C-[N,O]);!$(C12=CC(=O)CCC1C3C(C4C(CCC4)CC3)CC2)] |
| aryl_methyl | [$(a-[CH3]),$(a-[CH2]-[CH3]),$(a-[CH2]-[CH2]~[!N;!O]);!$(a(:a!:*):a!:*)] |
| Ndealkylation1 | [$(N(-[CH3])-C-[$(C~O),$(C-a),$(C-N),$(C=C)]),$(N(-[CH2][CH3])-C-[$(C~O),$(C-a),$(C-N),$(C=C)])] |
| Ndealkylation2 | [$([N&R1]1(-C)CCC1),$([N&R1]1(-C)CCCC1),$([N&R1]1(-C)CCCCC1),$([N&R1]1(-C)CCCCCC1),$([N&R1]1(-C)CCCCCCC1)] |
| alkyl_carbamate | C[NH1]C(=O)OC |
| ketone_Topliss | [$([CX3](=[OX1])(C)([c,C]));!$([CX3](=[OX1])([CH1]=C)[c,C])] |
| ArN | [$(a-[NX3H2]),$(a-[NH1][NH2]),$(a-C(=[OX1])[NH1][NH2]),$(a-C(=[NH])[NH2])] |
| HOCCN | [$([OX2H1][CX4][CX4H2][NX3&R1]),$([OH1][CX4][CX4H2][NX3][CX4](C)(C)C)] |
| AcidChloride | C(=O)Cl |
| AcidChloride.Aromatic | [$(C-!@[a])](=O)(Cl) |
| AcidChloride.Aliphatic | [$(C-!@[A;!Cl])](=O)(Cl) |
| CarboxylicAcid | C(=O)[O;H,-] |
| CarboxylicAcid.Aromatic | [$(C-!@[a])](=O)([O;H,-]) |

| | |
|---|---|
| CarboxylicAcid.Aliphatic | [$(C-!@[A;!O])](=O)([O;H,-]) |
| CarboxylicAcid.AlphaAmino | [$(C-[C;!$(C=[!#6])]-[N;!H0;!$(N-[!#6;!#1]);!$(N-C=[O,N,S])])](=O)([O;H,-]) |
| SulfonylChloride | [$(S-!@[#6])](=O)(=O)(Cl) |
| SulfonylChloride.Aromatic | [$(S-!@c)](=O)(=O)(Cl) |
| SulfonylChloride.Aliphatic | [$(S-!@C)](=O)(=O)(Cl) |
| Amine | [N;$(N-[#6]);!$(N-[!#6;!#1]);!$(N-C=[O,N,S])] |
| Amine.Primary | [N;H2;D1;$(N-!@[#6]);!$(N-C=[O,N,S])] |
| Amine.Primary.Aromatic | [N;H2;D1;$(N-!@c);!$(N-C=[O,N,S])] |
| Amine.Primary.Aliphatic | [N;H2;D1;$(N-!@C);!$(N-C=[O,N,S])] |
| Amine.Secondary | [N;H1;D2;$(N(-[#6])-[#6]);!$(N-C=[O,N,S])] |
| Amine.Secondary.Aromatic | [N;H1;D2;$(N(-[c])-[#6]);!$(N-C=[O,N,S])] |
| Amine.Secondary.Aliphatic | [N;H1;D2;$(N(-C)-C);!$(N-C=[O,N,S])] |
| Amine.Tertiary | [N;H0;D3;$(N(-[#6])(-[#6])-[#6]);!$(N-C=[O,N,S])] |
| Amine.Tertiary.Aromatic | [N;H0;D3;$(N(-[c])(-[#6])-[#6]);!$(N-C=[O,N,S])] |
| Amine.Tertiary.Aliphatic | [N;H0;D3;$(N(-C)(-C)-C);!$(N-C=[O,N,S])] |
| Amine.Aromatic | [N;$(N-c);!$(N-[!#6;!#1]);!$(N-C=[O,N,S])] |
| Amine.Aliphatic | [N;!$(N-c);$(N-C);!$(N-[!#6;!#1]);!$(N-C=[O,N,S])] |
| Amine.Cyclic | [N;R;$(N-[#6]);!$(N-[!#6;!#1]);!$(N-C=[O,N,S])] |
| BoronicAcid | [$(B-!@[#6])](O)(O) |
| BoronicAcid.Aromatic | [$(B-!@c)](O)(O) |
| BoronicAcid.Aliphatic | [$(B-!@C)](O)(O) |
| Isocyanate | [$(N-!@[#6])](=!@C=!@O) |
| Isocyanate.Aromatic | [$(N-!@c)](=!@C=!@O) |
| Isocyanate.Aliphatic | [$(N-!@C)](=!@C=!@O) |
| Alcohol | [O;H1;$(O-!@[#6;!$(C=!@[O,N,S])])] |
| Alcohol.Aromatic | [O;H1;$(O-!@c)] |
| Alcohol.Aliphatic | [O;H1;$(O-!@[C;!$(C=!@[O,N,S])])] |
| Aldehyde | [CH;D2;!$(C-[!#6;!#1])]=O |
| Aldehyde.Aromatic | [CH;D2;$(C-!@[a])](=O) |
| Aldehyde.Aliphatic | [CH;D2;$(C-!@C)](=O) |
| Halogen | [$([F,Cl,Br,I]-!@[#6]);!$([F,Cl,Br,I]-!@C-!@[F,Cl,Br,I]);!$([F,Cl,Br,I]-[C,S](=[O,S,N]))] |
| Halogen.Aromatic | [F,Cl,Br,I;$(*-!@c)] |
| Halogen.Aliphatic | [$([F,Cl,Br,I]-!@C);!$([F,Cl,Br,I]-!@C-!@[F,Cl,Br,I])] |
| Halogen.NotFluorine | [$([Cl,Br,I]-!@[#6]);!$([Cl,Br,I]-!@C-!@[F,Cl,Br,I]);!$([Cl,Br,I]-[C,S](=[O,S,N]))] |
| Halogen.NotFluorine.Aliphatic | [$([Cl,Br,I]-!@C);!$([Cl,Br,I]-!@C-!@[F,Cl,Br,I]);!$([Cl,Br,I]-[C,S](=[O,S,N]))] |
| Halogen.NotFluorine.Aromatic | [$([Cl,Br,I]-!@c)] |
| Halogen.Bromine | [Br;$([Br]-!@[#6]);!$([Br]-!@C-!@[F,Cl,Br,I]);!$([Br]-[C,S](=[O,S,N]))] |
| Halogen.Bromine.Aliphatic | [Br;$(Br-!@C);!$(Br-!@C-!@[F,Cl,Br,I]);!$(Br-[C,S](=[O,S,N]))] |
| Halogen.Bromine.Aromatic | [Br;$(Br-!@c)] |
| Halogen.Bromine.BromoKetone | [Br;$(Br-[CH2]-C(=O)-[#6])] |
| Azide | [N;H0;$(N-[#6]);D2]=[N;D2]=[N;D1] |
| Azide.Aromatic | [N;H0;$(N-c);D2]=[N;D2]=[N;D1] |

| | |
|---|---|
| Azide.Aliphatic | [N;H0;$(N-C);D2]=[N;D2]=[N;D1] |
| Nitro | [N;H0;$(N-[#6]);D3](=[O;D1])~[O;D1] |
| Nitro.Aromatic | [N;H0;$(N-c);D3](=[O;D1])~[O;D1] |
| Nitro.Aliphatic | [N;H0;$(N-C);D3](=[O;D1])~[O;D1] |
| TerminalAlkyne | [C;$(C#[CH])] |
| $-NC(=O)CH_3$ | *-[N;D2]-[C;D3](=O)-[C;D1;H3] |
| -C(=O)O | *-C(=O)[O;D1] |
| -C(=O)OMe | *-C(=O)[O;D2]-[C;D1;H3] |
| -C(=O)H | *-C(=O)-[C;D1] |
| -C(=O)N | *-C(=O)-[N;D1] |
| $-C(=O)CH_3$ | *-C(=O)-[C;D1;H3] |
| -N=C=O | *-[N;D2]=[C;D2]=[O;D1] |
| -N=C=S | *-[N;D2]=[C;D2]=[S;D1] |
| $-NO_2$ | *-[N;D3](=[O;D1])[O;D1] |
| -N=O | *-[N;R0]=[O;D1] |
| =N-O | *=[N;R0]-[O;D1] |
| $=NCH_3$ | *=[N;R0]-[C;D1;H3] |
| $-N=CH_2$ | *-[N;R0]=[C;D1;H2] |
| $-N=NCH_3$ | *-[N;D2]=[N;D2]-[C;D1;H3] |
| -N=N | *-[N;D2]=[N;D1] |
| -N#N | *-[N;D2]#[N;D1] |
| -C#N | *-[C;D2]#[N;D1] |
| $-SO_2NH_2$ | *-[S;D4](=[O;D1])(=[O;D1])-[N;D1] |
| $-NHSO_2CH_3$ | *-[N;D2]-[S;D4](=[O;D1])(=[O;D1])-[C;D1;H3] |
| $-SO_3H$ | *-[S;D4](=O)(=O)-[O;D1] |
| $-SO_3CH_3$ | *-[S;D4](=O)(=O)-[O;D2]-[C;D1;H3] |
| $-SO_2CH_3$ | *-[S;D4](=O)(=O)-[C;D1;H3] |
| $-SO_2Cl$ | *-[S;D4](=O)(=O)-[Cl] |
| $-SOCH_3$ | *-[S;D3](=O)-[C;D1] |
| $-SCH_3$ | *-[S;D2]-[C;D1;H3] |
| -S | *-[S;D1] |
| =S | *=[S;D1] |
| -X | *-[#9,#17,#35,#53] |
| -tBu | *-[C;D4]([C;D1])([C;D1])-[C;D1] |
| -CF3 | *-[C;D4](F)(F)F |
| -C#CH | *-[C;D2]#[C;D1;H] |
| -cPropyl | *-[C;D3]1-[C;D2]-[C;D2]1 |
| -OEt | *-[O;D2]-[C;D2]-[C;D1;H3] |
| -OMe | *-[O;D2]-[C;D1;H3] |
| -O | *-[O;D1] |
| =O | *=[O;D1] |
| -N | *-[N;D1] |
| =N | *=[N;D1] |
| #N | *#[N;D1] |
| Acid halide | C(=O)[Cl,Br,I,F] |
| Acyl cyanide | N#CC(=O) |
| Aldehyde | [CH1](=O) |
| Alkyl halide | [CX4][Cl,Br,I] |

| | |
|---|---|
| Azido group | N=[N+]=[N-] |
| Azo group | N#N |
| Azocane | [CH2R2]1N[CH2R2][CH2R2][CH2R2][CH2R2][CH2R2][CH2R2]1 |
| Benzidine | [cR2]1[cR2][cR2]([Nv3X3,Nv4X4])[cR2][cR2][cR2]1[cR2]2[cR2][cR2][cR2]([Nv3X3,Nv4X4])[cR2][cR2]2 |
| Betaketo/anhydride | [C,c](=O)[CX4,CR0X3,O][C,c](=O) |
| Biotin analogue | C12C(NC(N1)=O)CSC2 |
| Carbo cation/anion | [C+,c+,C-,c-] |
| Catechol | c1c([OH])c([OH,NH2,NH])ccc1 |
| Charged oxygen or sulfur atoms | [O+,o+,S+,s+] |
| Chinone | C1(=[O,N])C=CC(=[O,N])C=C1 |
| Chinone_2 | C1(=[O,N])C(=[O,N])C=CC=C1 |
| Conjugated nitrile group | C=[C!r]C#N |
| Crown ether | [OR2,NR2]@[CR2]@[CR2]@[OR2,NR2]@[CR2]@[CR2]@[OR2,NR2] |
| Cumarine | c1ccc2c(c1)ccc(=O)o2 |
| Cyanamide | N[CH2]C#N |
| Cyanate/aminonitrile/thiocyanate | [N,O,S]C#N |
| Cyanohydrins | N#CC[OH] |
| Diaminobenzene | [cR2]1[cR2]c([N+0X3R0,nX3R0])c([N+0X3R0,nX3R0])[cR2][cR2]1 |
| Diaminobenzene_2 | [cR2]1[cR2]c([N+0X3R0,nX3R0])[cR2]c([N+0X3R0,nX3R0])[cR2]1 |
| Diaminobenzene_3 | [cR2]1[cR2]c([N+0X3R0,nX3R0])[cR2][cR2]c1([N+0X3R0,nX3R0]) |
| Diazo group | [N!R]=[N!R] |
| Diketo group | [C,c](=O)[C,c](=O) |
| Disulphide | SS |
| Ester of HOBT | C(=O)Onnn |
| Four member lactones | C1(=O)OCC1 |
| Heavy metal | [Hg,Fe,As,Sb,Zn,Se,se,Te,Si] |
| Het-C-het not in ring | [NX3R0,NX4R0,OR0,SX2R0][CX4][NX3R0,NX4R0,OR0,SX2R0] |
| Hydrazine | N[NH2] |
| Hydroquinone | [OH]c1ccc([OH,NH2,NH])cc1 |
| Iodine | I |
| Ketene | C=C=O |
| Methylidene-1,3-dithiole | S1C=CSC1=S |
| Michael acceptor | C=!@CC=[O,S] |
| Michael acceptor_2 | [$([CH]),$(CC)]#CC(=O)[C,c] |
| Michael acceptor_3 | [$([CH]),$(CC)]#CS(=O)(=O)[C,c] |
| Michael acceptor_4 | C=C(C=O)C=O |
| Michael acceptor_5 | [$([CH]),$(CC)]#CC(=O)O[C,c] |
| N oxide | [NX2,nX3][OX1] |
| N-acyl-2-amino-5-mercapto-1,3,4-thiadiazole | s1c(S)nnc1NC=O |
| N-C-halo | NC[F,Cl,Br,I] |

| | |
|---|---|
| N-halo | [NX3,NX4][F,Cl,Br,I] |
| N-hydroxyl pyridine | n[OH] |
| N-nitroso | [#7]-N=O |
| Perfluorinated chain | [CX4](F)(F)[CX4](F)F |
| Peroxide | OO |
| Phenol ester | c1ccccc1OC(=O)[#6] |
| Phenyl carbonate | c1ccccc1OC(=O)O |
| Phosphor | P |
| Polyene | [CR0]=[CR0][CR0]=[CR0] |
| Quaternary nitrogen | [s,S,c,C,n,N,o,O]~[nX3+,NX3+](~[s,S,c,C,n,N])~[s,S,c,C,n,N] |
| Quaternary nitrogen_2 | [s,S,c,C,n,N,o,O]~[n+,N+](~[s,S,c,C,n,N,o,O])(~[s,S,c,C,n,N,o,O])~[s,S,c,C,n,N,o,O] |
| Quaternary nitrogen_3 | [*]=[N+]=[*] |
| Saponine derivative | O1CCCCC1OC2CCC3CCCCC3C2 |
| Silicon halogen | [Si][F,Cl,Br,I] |
| Stilbene | c1ccccc1C=Cc2ccccc2 |
| Sulfinic acid | [SX3](=O)[O-,OH] |
| Sulfonic acid_2 | [C,c]S(=O)(=O)O[C,c] |
| Sulfonic acid_3 | S(=O)(=O)[O-,OH] |
| Sulfonyl cyanide | S(=O)(=O)C#N |
| Sulfur oxygen single bond | [SX2]O |
| Sulphate | OS(=O)(=O)[O-] |
| Sulphur nitrogen single bond | [SX2H0][N] |
| Thiobenzothiazole | c12ccccc1(SC(S)=N2) |
| Thiobenzothiazole_2 | c12ccccc1(SC(=S)N2) |
| Thiocarbonyl group | [C,c]=S |
| Thioester | SC=O |
| Thiol | [S-] |
| Three-membered heterocycle | *1[O,S,N]*1 |
| Triflate | OS(=O)(=O)C(F)(F)F |
| Triphenyl methylsilyl | [SiR0,CR0](c1ccccc1)(c2ccccc2)(c3ccccc3) |

# Compound characterization

## General section

Compounds were obtained from commercial sources (i.e., Acros Organics, Apollo Scientific, BLD Pharmatech, Fluorochem, MedChemExpress, Merck, TargetMol, TCI Europe) and used as received. $^1$H and $^{13}$C NMR spectra were recorded in a deuterated solvent on a Bruker Avance III 400 MHz spectrometer, operating at 400 and 101 MHz frequency. NMR spectra were measured at room temperature (25 °C) in deuterated DMSO. Chemical shifts (δ) are expressed in *parts per million* (*ppm*) referenced to TMS or residual solvent signals. Spectral data are reported in the following format: chemical shift (multiplicity, coupling constants, number of hydrogens). All coupling constants (*J*) are reported in Hertz. Compound purity was determined by HPLC analysis on Thermo Scientific Dionex UltiMate 3000 modular system (Thermo Fisher Scientific Inc.) with Waters Acquity UPLC® HSS C18 SB column (2.1 × 50 mm, 1.8 µm) thermostated at 40 °C, injection volume, 1 µL; flow rate, 0.3 mL/min; detector λ, 254 nm; mobile phase A: 0.1% TFA (v/v) in water; mobile phase B: MeCN.

Method: 0–5 min, 5%–30% B; 5-9 min, 100% B; 9-10 min, 100% B. Compounds are >95% pure by HPLC analysis, unless stated otherwise

## Analytical data and chromatograms

**Fisetin** (**2**)

$^{1}H$ NMR (400 MHz, DMSO-$d_6$): δ 11.33 – 10.00 (bs, 1H), 9.97 – 8.38 (m, 3H), 7.93 (d, $J$ = 9.4 Hz, 1H), 7.70 (d, $J$ = 2.2 Hz, 1H), 7.55 (dd, $J$ = 8.5, 2.2 Hz, 1H), 6.95 – 6.86 (m, 3H) ppm. $^{13}C$ NMR (101 MHz, DMSO-$d_6$): δ 172.1, 162.3, 156.4, 147.3, 145.1, 137.3, 126.6, 122.6, 119.7, 115.7, 115.0, 114.8, 114.3, 101.9 ppm. HPLC purity: 98.9 %

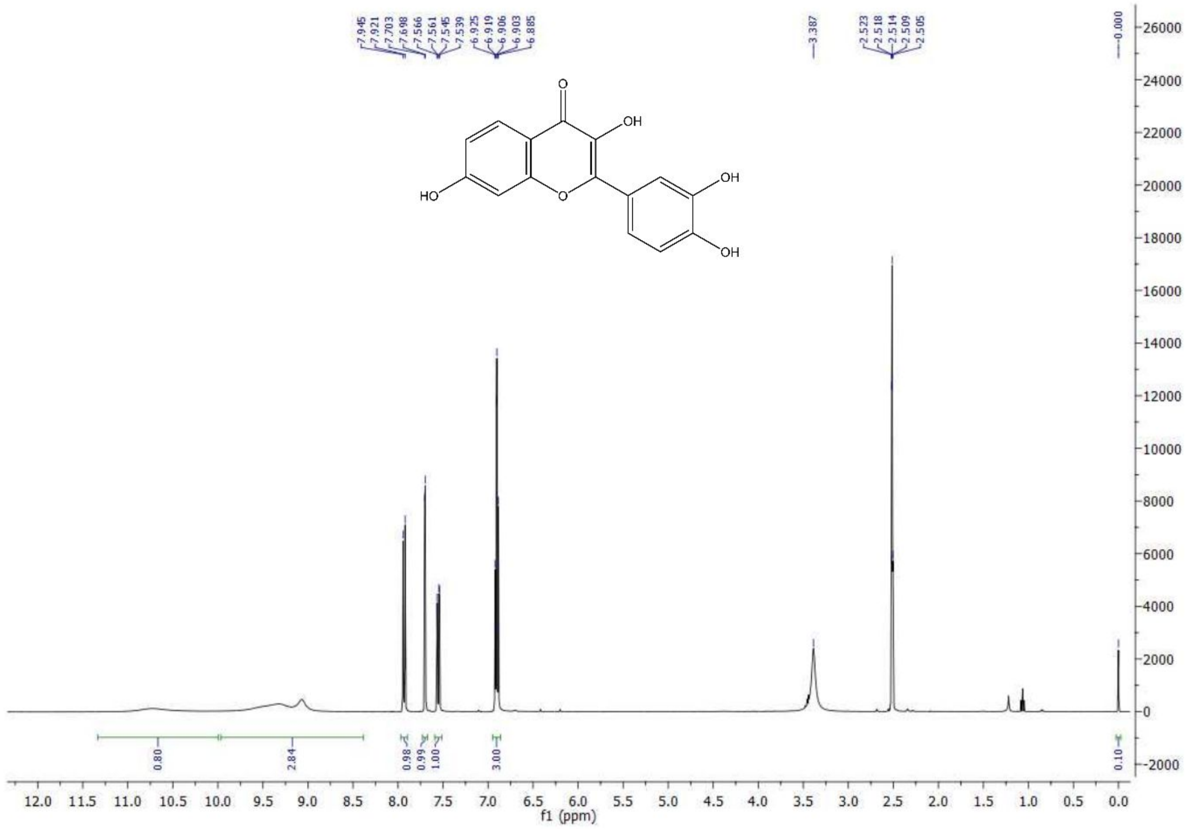


**Figure S2**. $^{1}H$-NMR spectrum of fisetin (**2**)

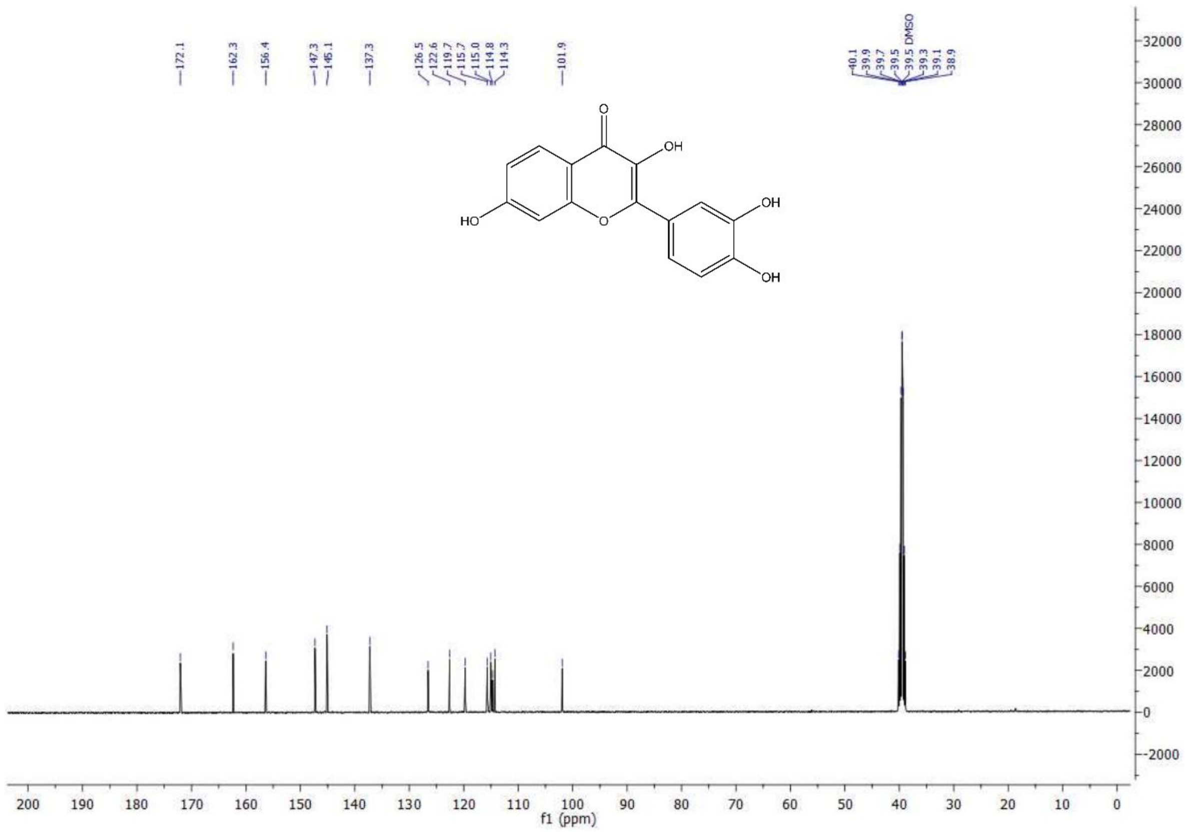


**Figure S3**. $^{13}$C-NMR spectrum of fisetin (**2**)

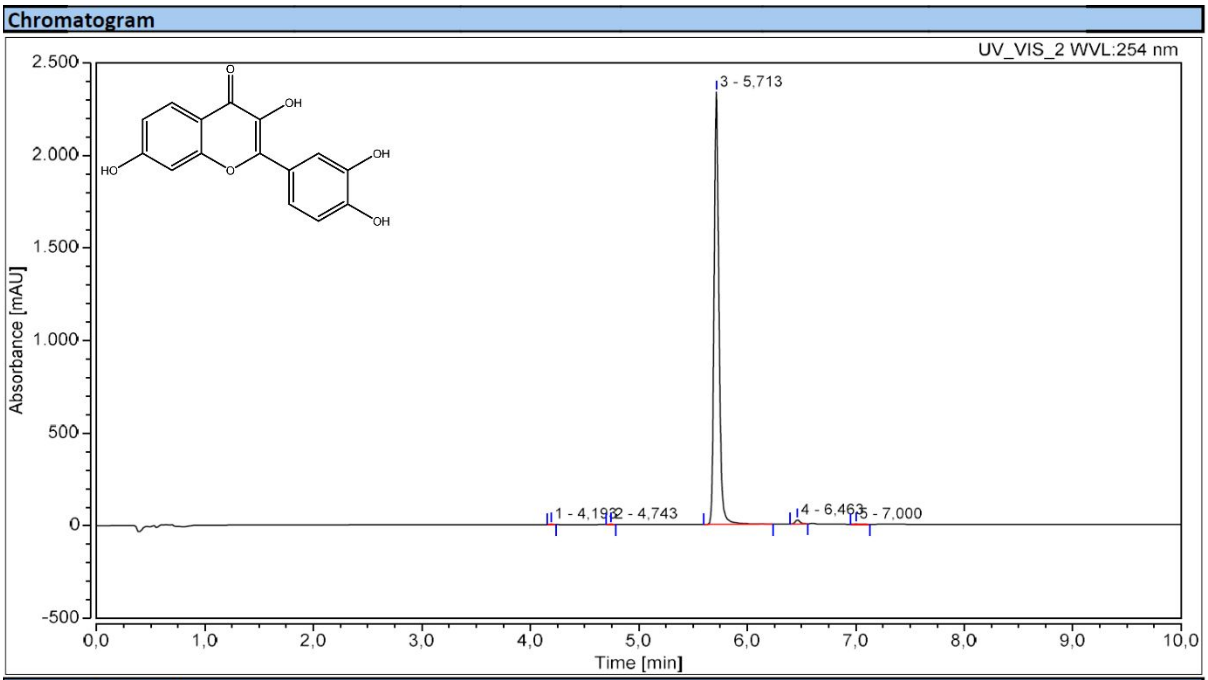


**Integration Results**

| No. | Peak Name | Retention Time min | Area mAU*min | Height mAU | Relative Area % | Relative Height % | Amount n.a. |
|---|---|---|---|---|---|---|---|
| 1 | | 4,193 | 0,084 | 2,111 | 0,07 | 0,09 | n.a. |
| 2 | | 4,743 | 0,069 | 1,430 | 0,06 | 0,06 | n.a. |
| 3 | | 5,713 | 121,510 | 2335,382 | 98,87 | 98,81 | n.a. |
| 4 | | 6,463 | 1,115 | 22,462 | 0,91 | 0,95 | n.a. |
| 5 | | 7,000 | 0,125 | 2,173 | 0,10 | 0,09 | n.a. |
| **Total:** | | | **122,904** | **2363,557** | **100,00** | **100,00** | |

**Figure S4**. HPLC chromatogram of fisetin (**2**)

**Myricetin (4)**

$^1$H NMR (400 MHz, DMSO-$d_6$): δ 12.51 (s, 1H), 10.80 (s, 1H), 9.37 (s, 1H), 9.24 (s, 2H), 8.83 (s, 1H), 7.25 (s, 2H), 6.38 (d, $J$ = 2.0 Hz, 1H), 6.19 (d, $J$ = 2.0 Hz, 1H) ppm. $^{13}$C NMR (101 MHz, DMSO-$d_6$): δ 175.8, 163.9, 160.7, 156.1, 146.8, 145.7, 135.9, 135.9, 120.8, 107.2, 103.0, 98.2, 93.2 ppm. HPLC purity: 99.6 %.

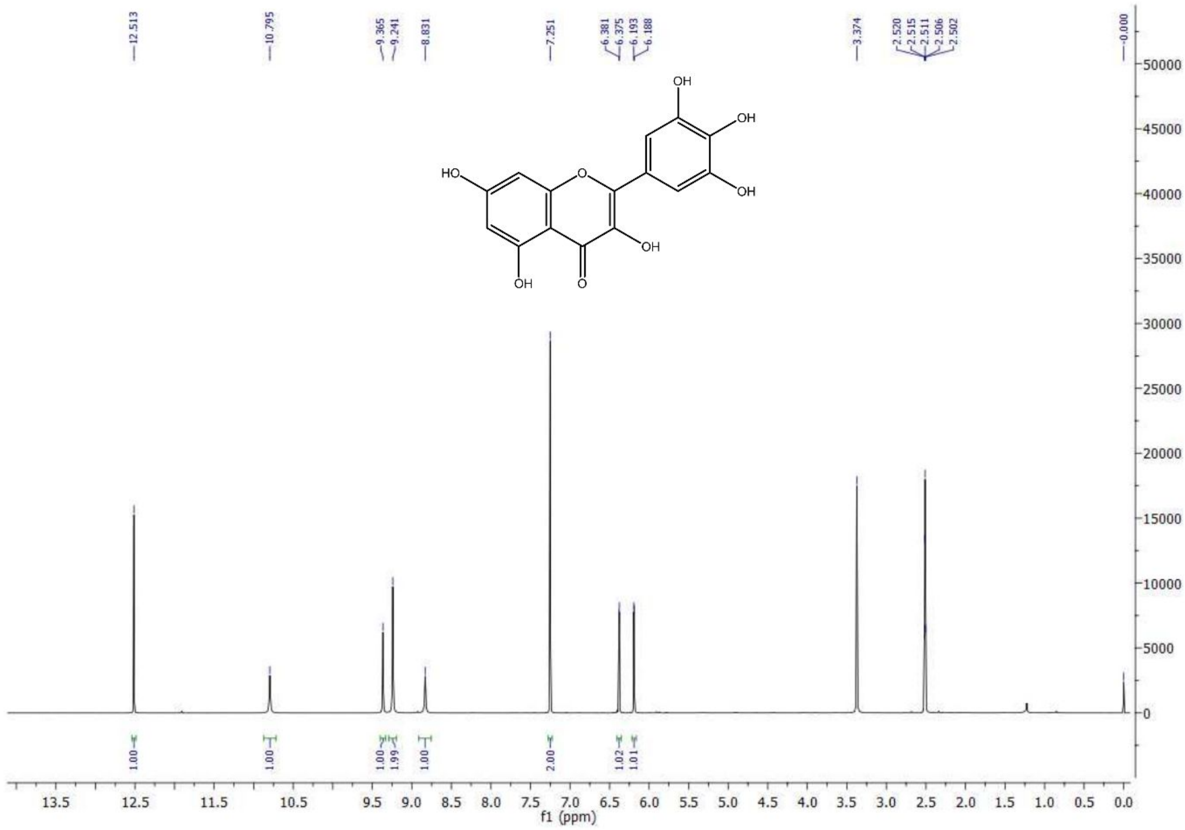


**Figure S5**. $^{1}$H-NMR spectrum of myricetin (**4**)

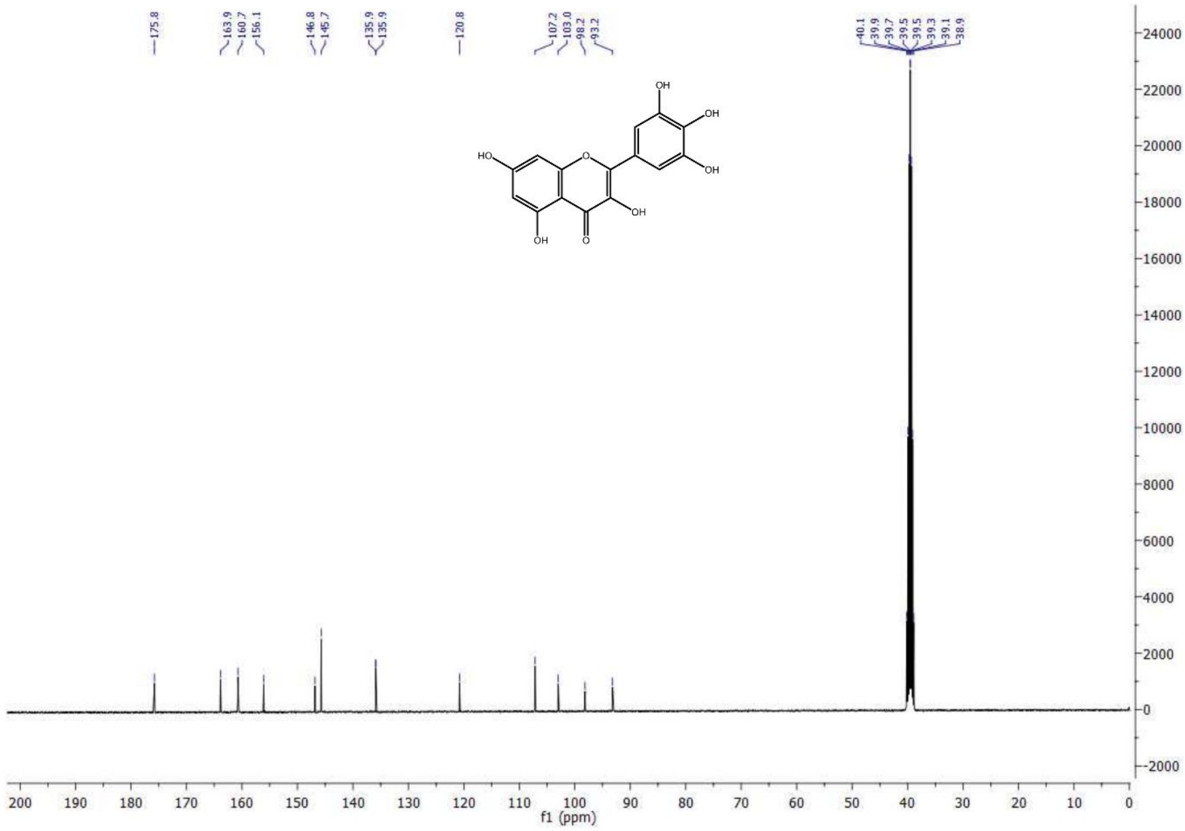


**Figure S6**. $^{13}$C-NMR spectrum of myricetin (**4**)

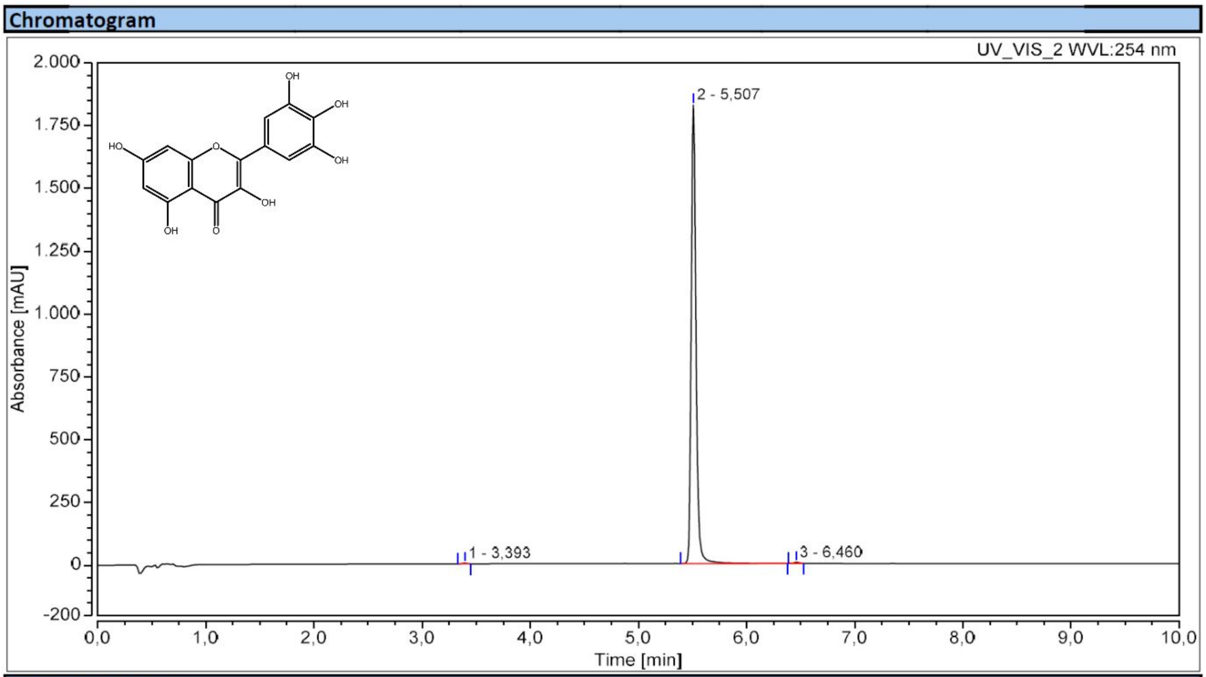


**Integration Results**

| No. | Peak Name | Retention Time min | Area mAU*min | Height mAU | Relative Area % | Relative Height % | Amount n.a. |
|---|---|---|---|---|---|---|---|
| 1 | | 3,393 | 0,151 | 3,134 | 0,16 | 0,17 | n.a. |
| 2 | | 5,507 | 94,011 | 1825,486 | 99,58 | 99,56 | n.a. |
| 3 | | 6,460 | 0,244 | 4,992 | 0,26 | 0,27 | n.a. |
| **Total:** | | | **94,405** | **1833,613** | **100,00** | **100,00** | |

**Figure S7**. HPLC chromatogram of myricetin (**4**)

**Quercetin** (**9**)

$^{1}$H NMR (400 MHz, DMSO-$d_6$): δ 12.50 (s, 3H), 10.79 (s, 3H), 9.61 (s, 3H), 9.39 (s, 4H), 9.32 (s, 2H), 7.68 (d, *J* = 2.2 Hz, 3H), 7.54 (dd, *J* = 8.5, 2.2 Hz, 3H), 6.89 (d, *J* = 8.5 Hz, 3H), 6.41 (d, *J* = 2.0 Hz, 3H), 6.19 (d, *J* = 2.0 Hz, 3H) ppm. $^{13}$C NMR (101 MHz, DMSO-$d_6$): δ 175.9, 163.9, 160.8, 156.2, 147.8, 146.8, 145.1, 135.8, 122.0, 120.0, 115.7, 115.1, 103.1, 98.2, 93.4 ppm. HPLC purity: 96.7

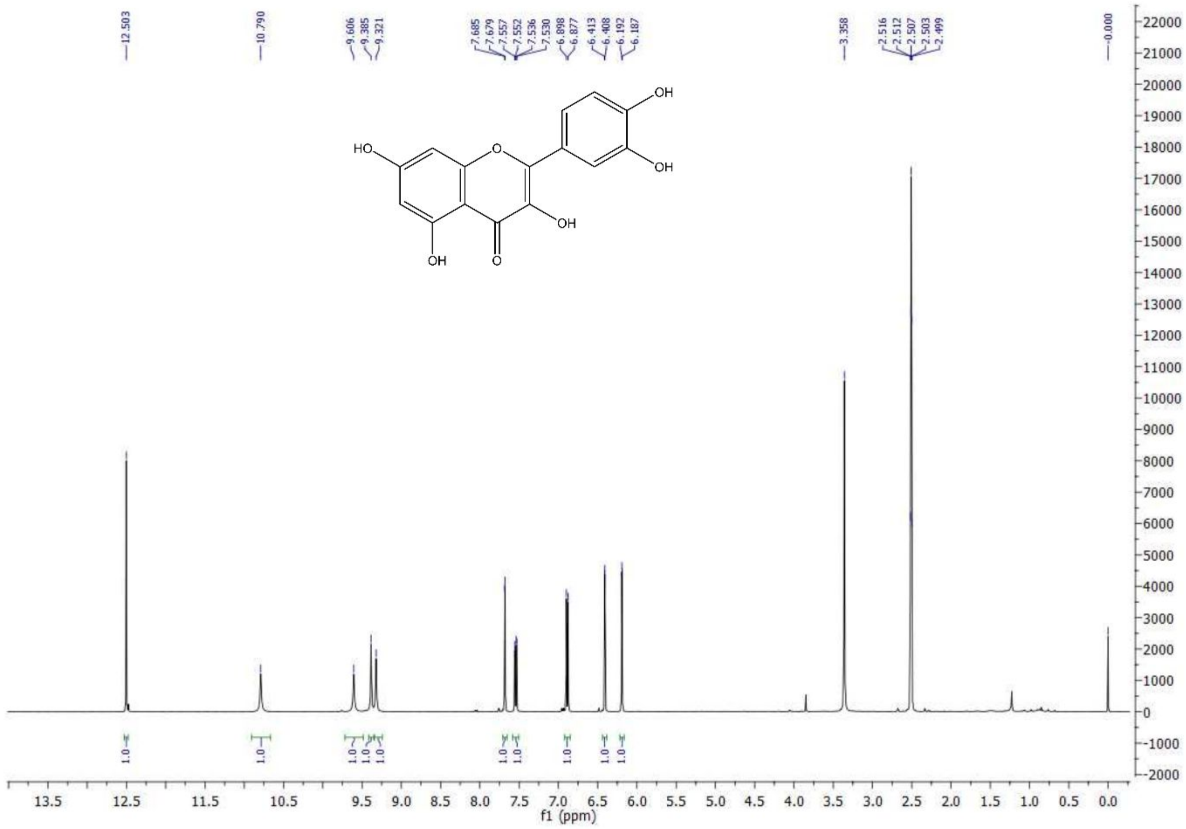

**Figure S8**. $^{1}$H-NMR spectrum of quercetin (**9**)

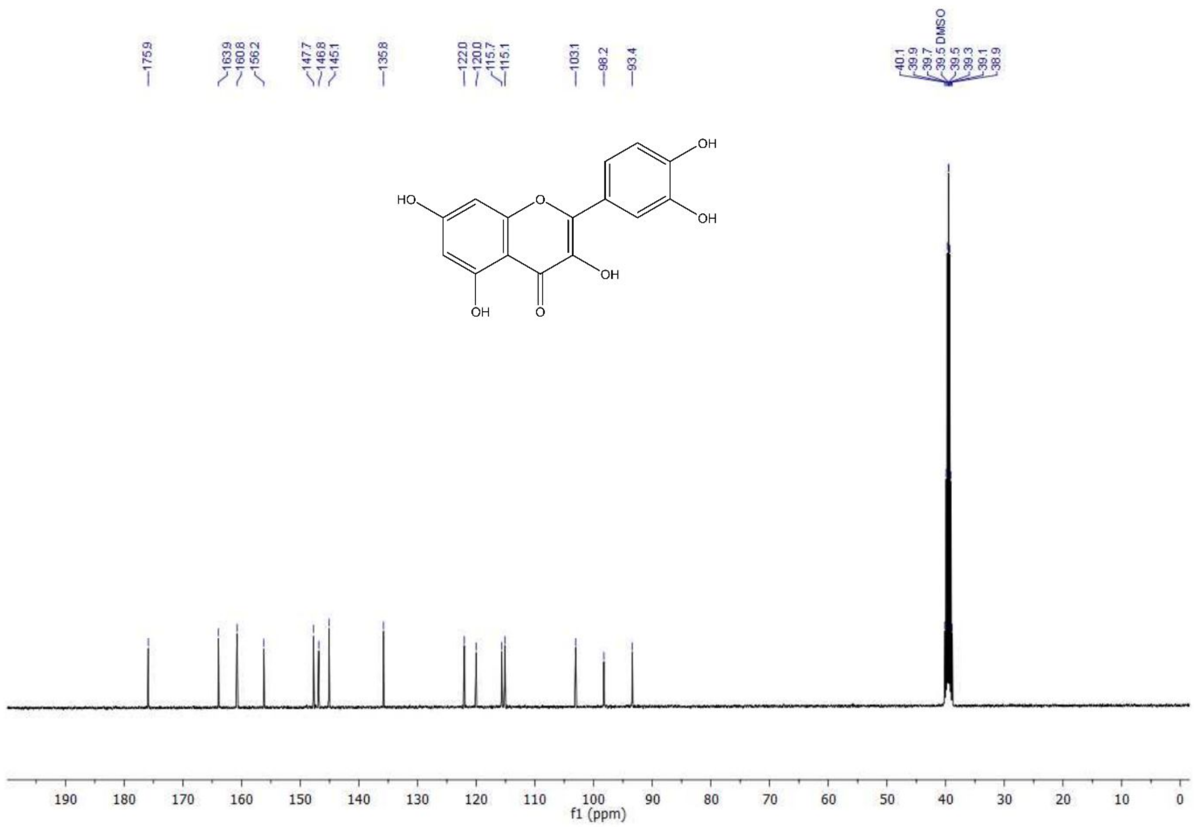

**Figure S9**. $^{13}$C-NMR spectrum of quercetin (**9**)

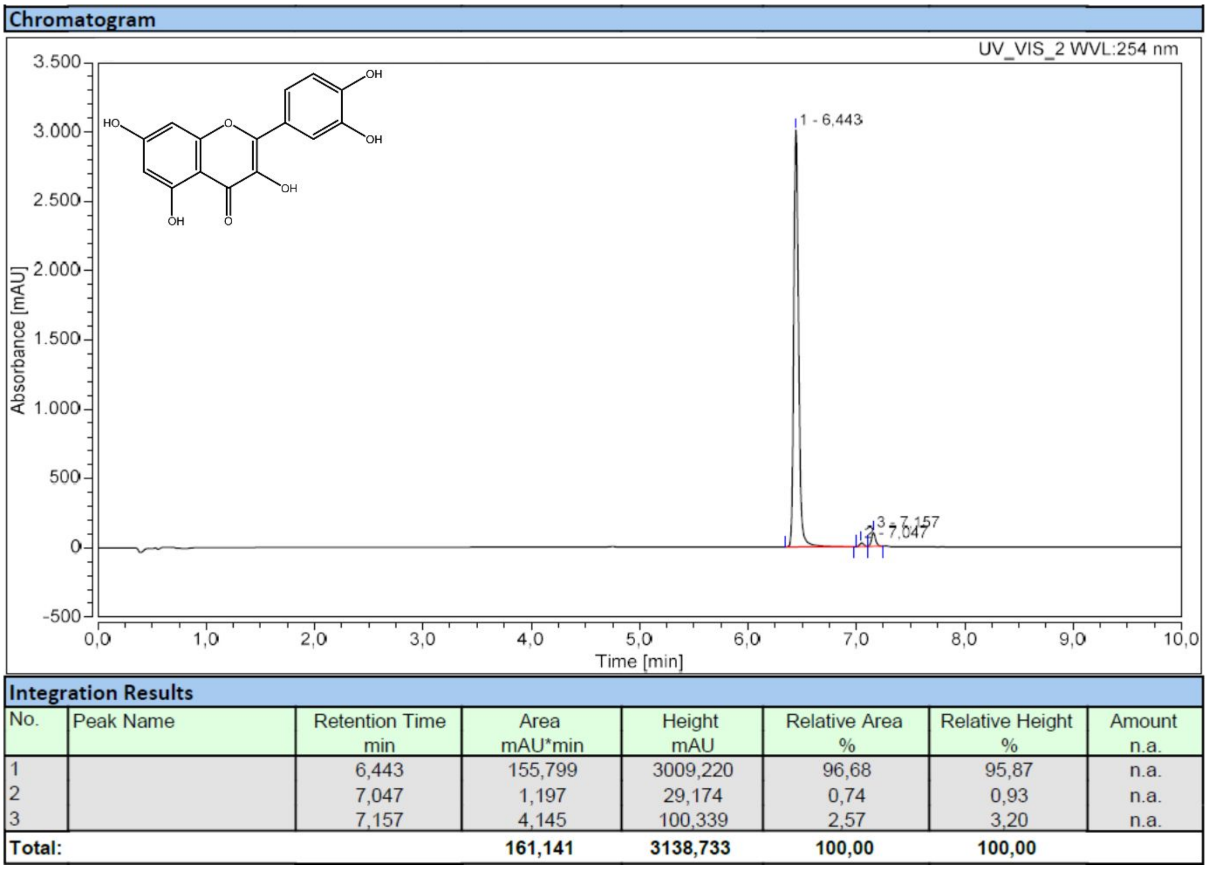


**Integration Results**

| No. | Peak Name | Retention Time min | Area mAU*min | Height mAU | Relative Area % | Relative Height % | Amount n.a. |
|---|---|---|---|---|---|---|---|
| 1 | | 6,443 | 155,799 | 3009,220 | 96,68 | 95,87 | n.a. |
| 2 | | 7,047 | 1,197 | 29,174 | 0,74 | 0,93 | n.a. |
| 3 | | 7,157 | 4,145 | 100,339 | 2,57 | 3,20 | n.a. |
| **Total:** | | | **161,141** | **3138,733** | **100,00** | **100,00** | |

**Figure S10**. HPLC chromatogram of quercetin (**9**)

**Quercitrin** (**10**)

$^{1}$H NMR (400 MHz, DMSO-d$_6$): δ 12.67 (s, 1H), 10.89 (s, 1H), 9.73 (s, 1H), 9.36 (s, 1H), 7.30 (d, J = 2.1 Hz, 1H), 7.26 (dd, $J$ = 8.3, 2.1 Hz, 1H), 6.87 (d, $J$ = 8.3 Hz, 1H), 6.40 (d, $J$ = 2.1 Hz, 1H), 6.21 (d, $J$ = 2.1 Hz, 1H), 5.26 (d, $J$ = 1.3 Hz, 1H), 4.96 (d, $J$ = 4.3 Hz, 1H), 4.74 (d, $J$ = 4.6 Hz, 1H), 4.63 (d, $J$ = 5.8 Hz, 1H), 3.98 (t, $J$ = 4.3 Hz, 1H), 3.51 (ddd, $J$ = 8.9, 5.6, 3.4 Hz, 1H), 3.27 – 3.07 (m, 2H), 0.82 (d, $J$ = 6.0 Hz, 3H) ppm. $^{13}$C NMR (101 MHz, DMSO-d$_6$): δ 177.8, 164.2, 161.3, 157.3, 156.5, 148.4, 145.2, 134.2, 121.1, 120.7, 115.6, 115.5, 104.1, 101.8, 98.7, 93.6, 71.2, 70.6, 70.3, 70.1, 17.5 ppm. HPLC purity: 99.7 %.

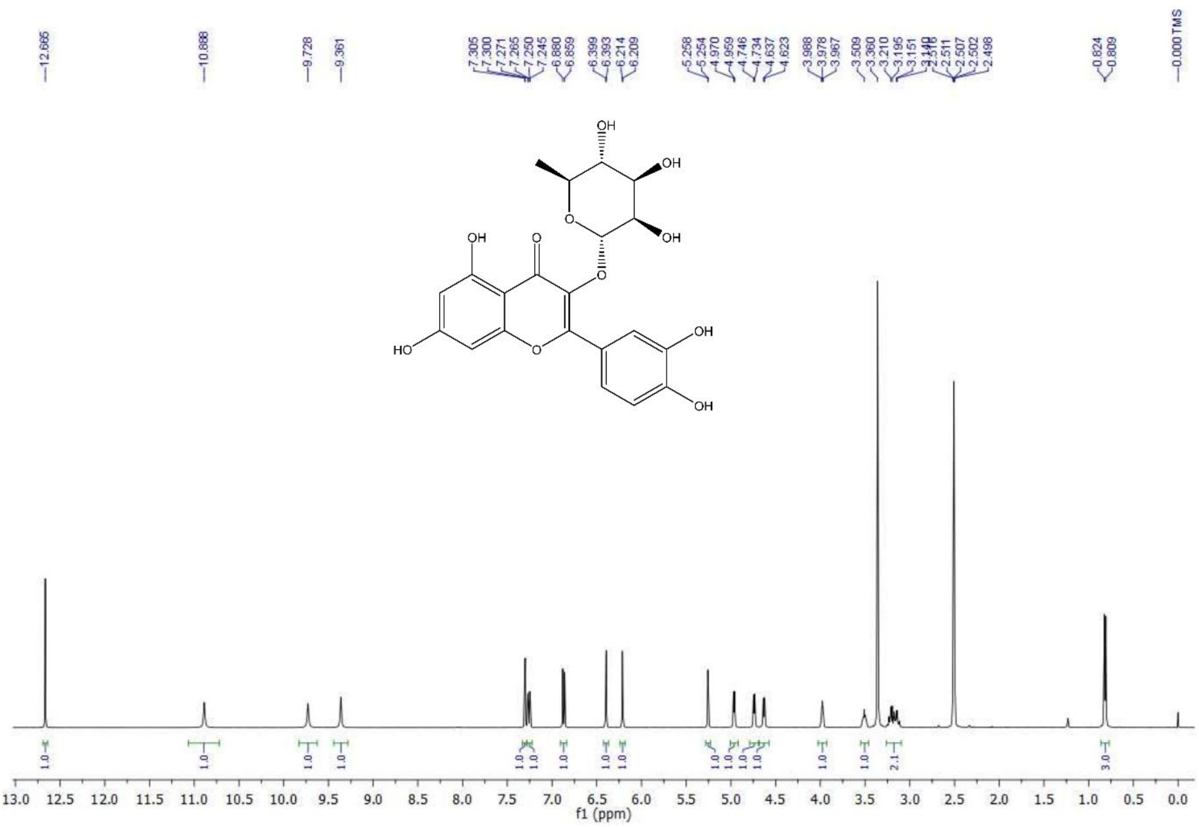

**Figure S11**. $^{1}$H-NMR spectrum of quercitrin (**10**)

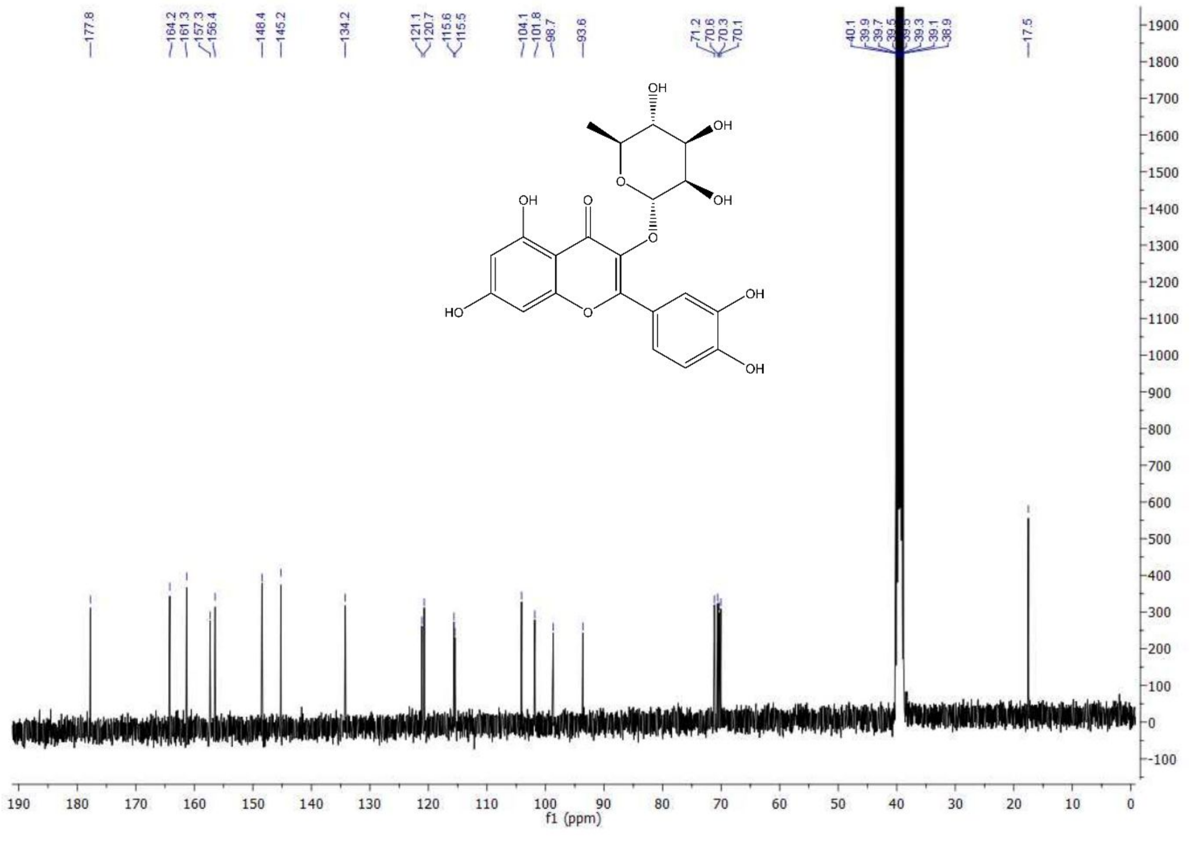

**Figure S12**. $^{13}$C-NMR spectrum of quercitrin (**10**)

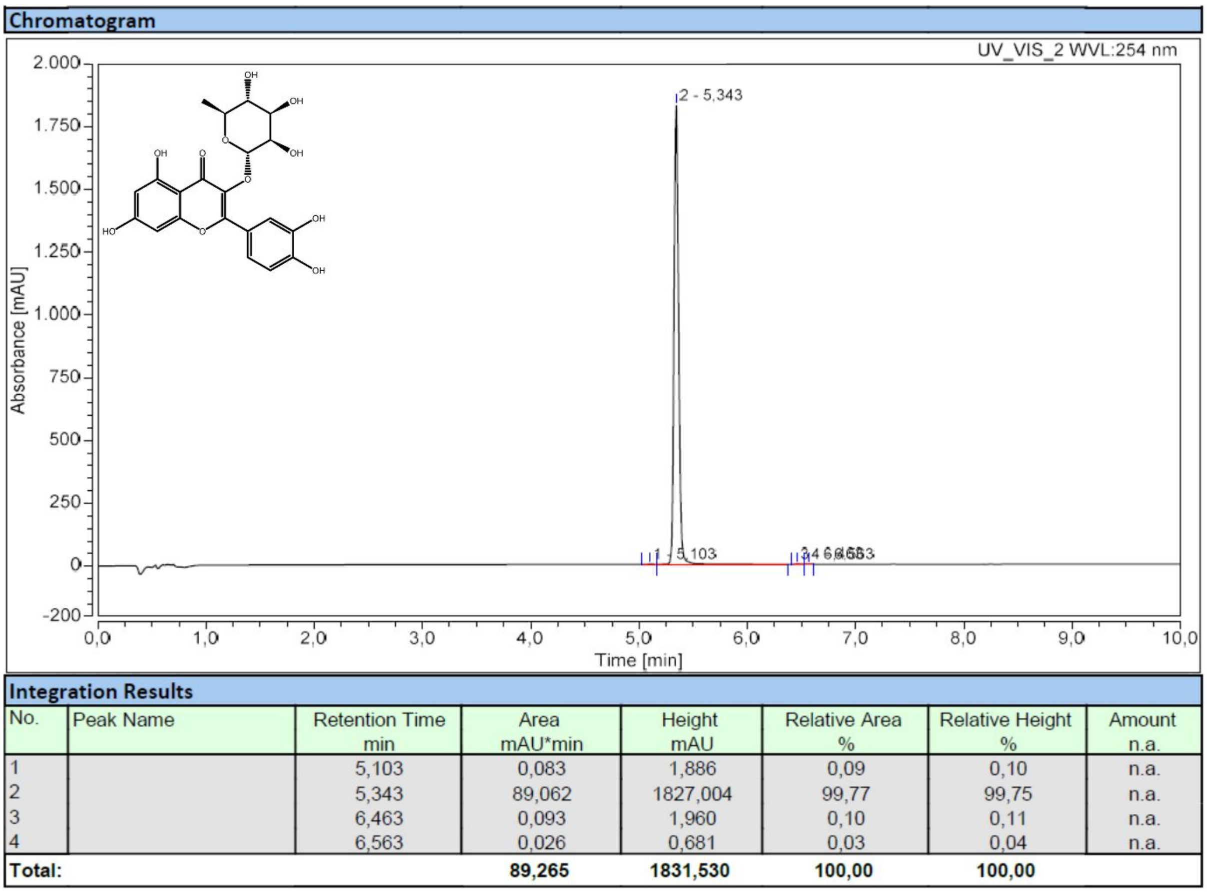


**Integration Results**

| No. | Peak Name | Retention Time min | Area mAU*min | Height mAU | Relative Area % | Relative Height % | Amount n.a. |
|---|---|---|---|---|---|---|---|
| 1 | | 5,103 | 0,083 | 1,886 | 0,09 | 0,10 | n.a. |
| 2 | | 5,343 | 89,062 | 1827,004 | 99,77 | 99,75 | n.a. |
| 3 | | 6,463 | 0,093 | 1,960 | 0,10 | 0,11 | n.a. |
| 4 | | 6,563 | 0,026 | 0,681 | 0,03 | 0,04 | n.a. |
| **Total:** | | | **89,265** | **1831,530** | **100,00** | **100,00** | |

**Figure S13**. HPLC chromatogram of quercitrin (**10**)

**Luteolin** (**17**)

$^{1}$H NMR (400 MHz, DMSO-$d_6$): δ 12.99 (s, 1H), 10.83 (s, 1H), 9.92 (s, 1H), 9.47 (s, 1H), 7.42 (d, *J* = 10.0 Hz, 2H), 6.90 (d, *J* = 8.2 Hz, 1H), 6.68 (s, 1H), 6.45 (d, *J* = 2.0 Hz, 1H), 6.20 (d, *J* = 2.0 Hz, 1H) ppm. $^{13}$C NMR (101 MHz, DMSO-$d_6$): δ 181.8, 164.2, 164.0, 161.6, 157.4, 149.8, 145.8, 121.7, 119.1, 116.1, 113.5, 103.8, 103.0, 98.9, 94.0 ppm. HPLC purity: 97.4%

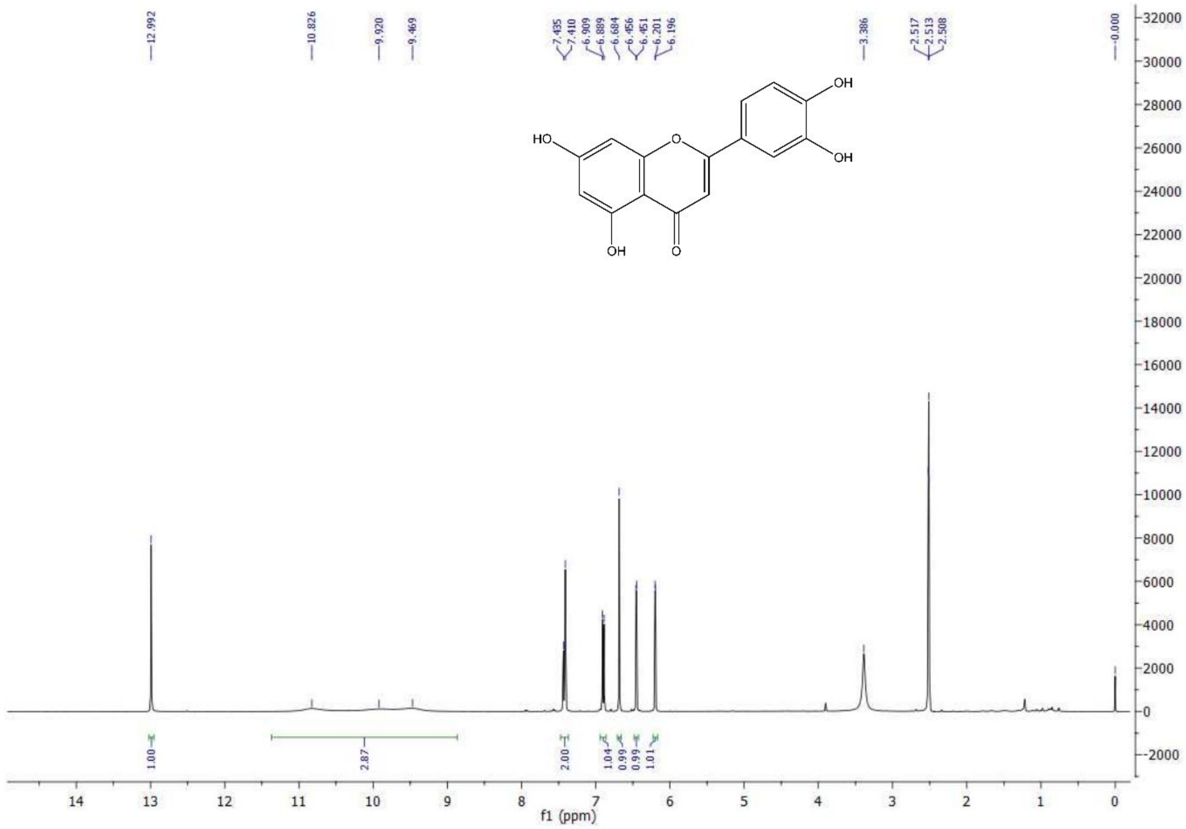


**Figure S14**. $^{1}$H-NMR spectrum of luteolin (**17**)

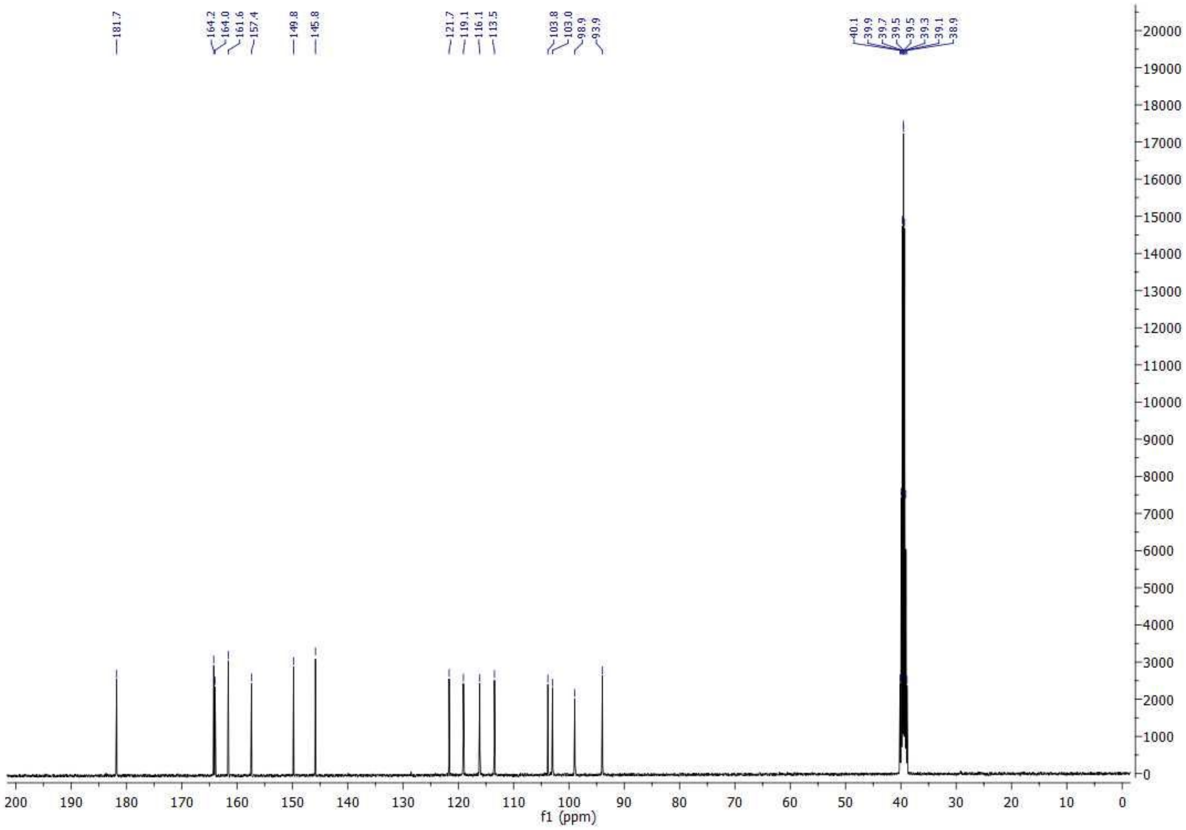


**Figure S15**. $^{13}$C-NMR spectrum of luteolin (**17**)

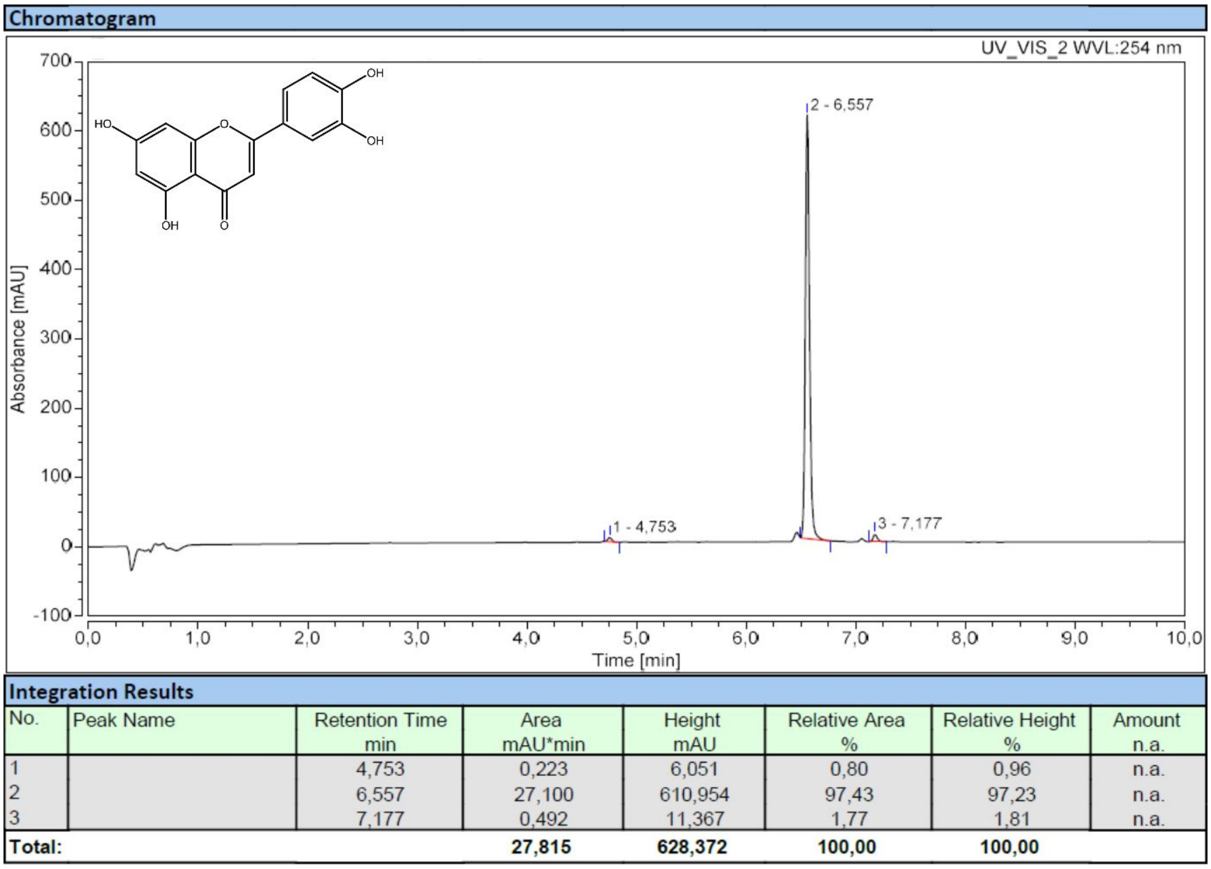


**Integration Results**

| No. | Peak Name | Retention Time min | Area mAU*min | Height mAU | Relative Area % | Relative Height % | Amount n.a. |
|---|---|---|---|---|---|---|---|
| 1 | | 4,753 | 0,223 | 6,051 | 0,80 | 0,96 | n.a. |
| 2 | | 6,557 | 27,100 | 610,954 | 97,43 | 97,23 | n.a. |
| 3 | | 7,177 | 0,492 | 11,367 | 1,77 | 1,81 | n.a. |
| **Total:** | | | **27,815** | **628,372** | **100,00** | **100,00** | |

**Figure S16**. HPLC chromatogram of luteolin (**17**)

**7,8-Dihydroxyflavone** (**25**)

$^{1}$H NMR (400 MHz, DMSO-d$_6$) δ 8.16 (dd, *J* = 6.6, 3.1 Hz, 2H), 7.63 – 7.51 (m, 3H), 7.41 (d, *J* = 8.7 Hz, 1H), 7.01 (d, *J* = 8.7 Hz, 1H), 6.91 (s, 1H) ppm. $^{13}$C NMR (101 MHz, DMSO-d$_6$): δ 177.1, 161.9, 150.7, 146.8, 133.3, 131.6, 129.2, 126.5, 117.1, 115.2, 114.4, 106.1 ppm. HPLC purity: 97.9 %

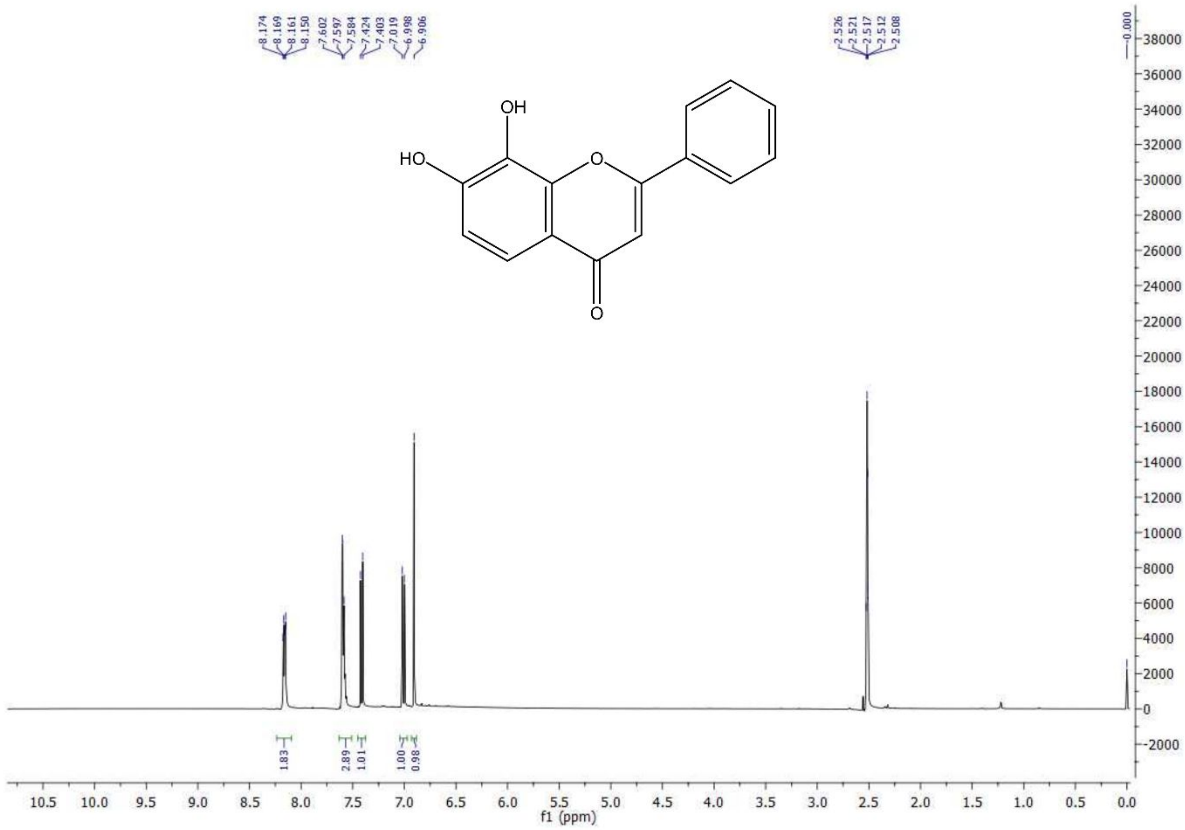


**Figure S17**. $^{3}$H-NMR spectrum of 7,8-dihydroxyflavone (**25**)

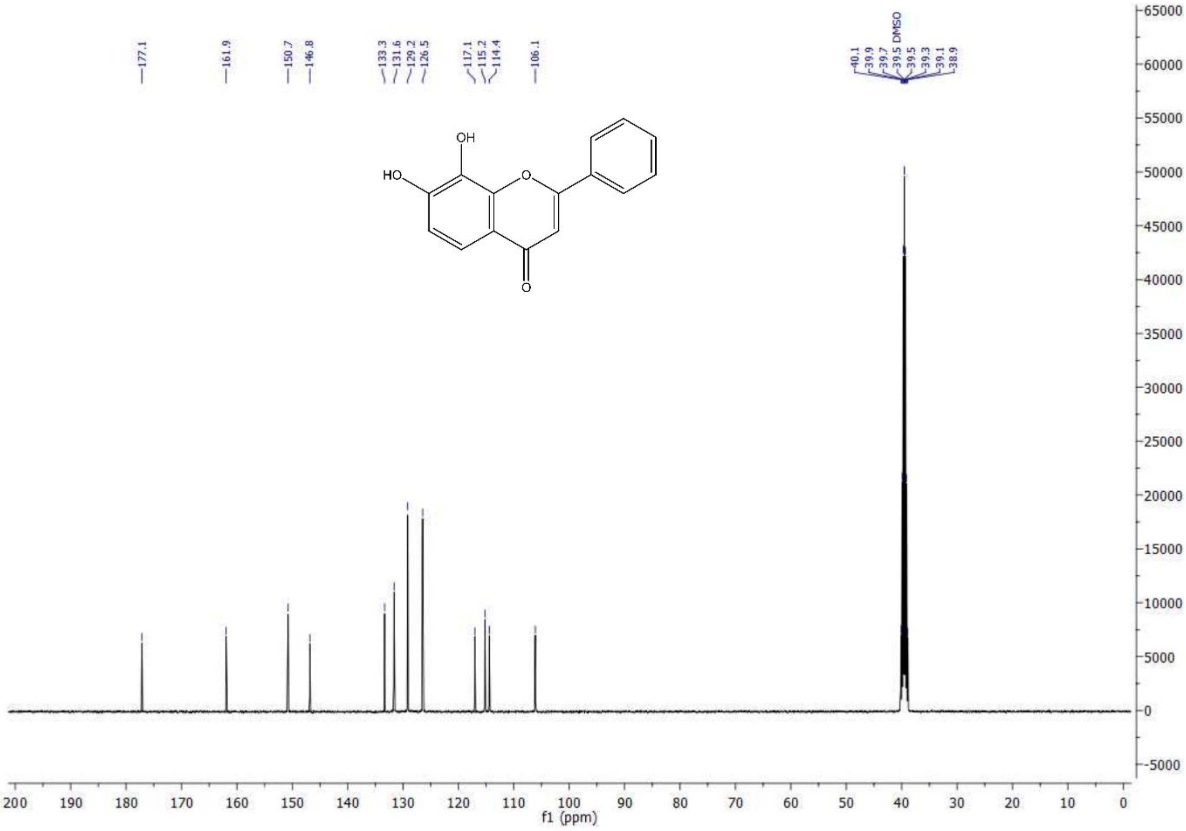


**Figure S18**. $^{13}$C-NMR spectrum of 7,8-dihydroxyflavone (**25**)

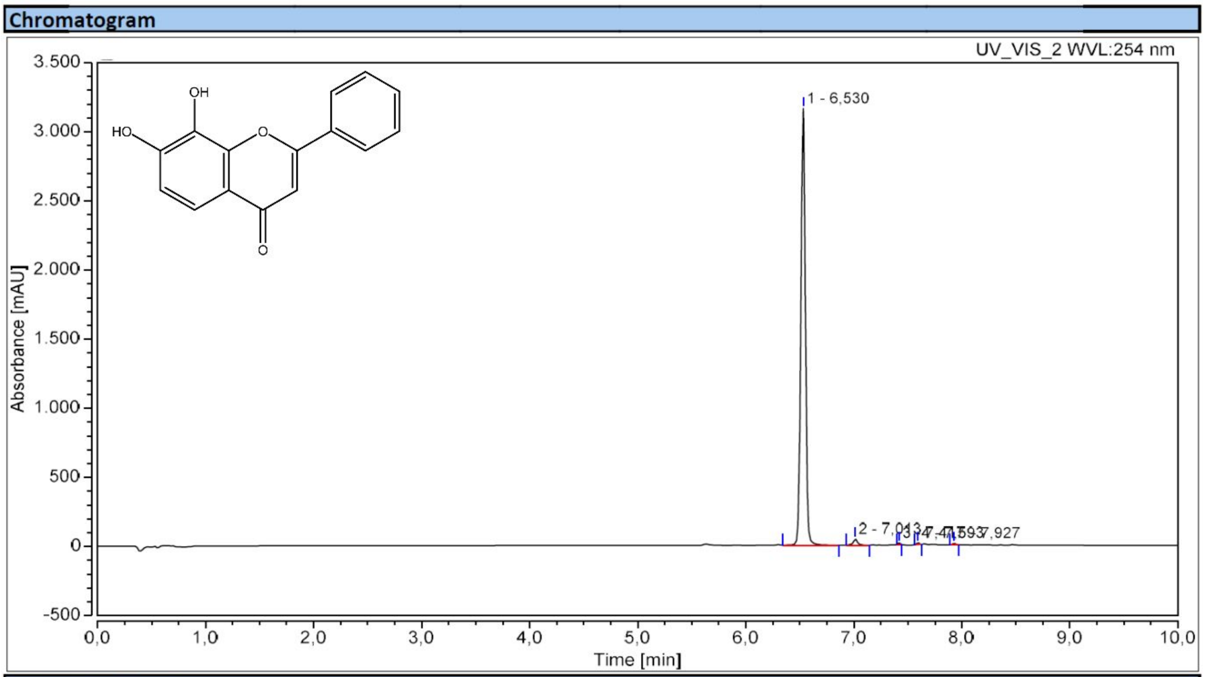


**Integration Results**

| No. | Peak Name | Retention Time min | Area mAU*min | Height mAU | Relative Area % | Relative Height % | Amount n.a. |
|---|---|---|---|---|---|---|---|
| 1 | | 6,530 | 153,038 | 3159,531 | 97,85 | 97,45 | n.a. |
| 2 | | 7,013 | 2,211 | 46,062 | 1,41 | 1,42 | n.a. |
| 3 | | 7,417 | 0,343 | 12,105 | 0,22 | 0,37 | n.a. |
| 4 | | 7,593 | 0,423 | 13,376 | 0,27 | 0,41 | n.a. |
| 5 | | 7,927 | 0,387 | 10,992 | 0,25 | 0,34 | n.a. |
| **Total:** | | | **156,401** | **3242,066** | **100,00** | **100,00** | |

**Figure S19**. HPLC chromatogram of 7,8-dihydroxyflavone (**25**)

**Baicalein** (**26**)

$^{1}$H NMR (400 MHz, DMSO-d$_6$): δ 12.73 (s, 1H), 10.63 (s, 1H), 8.89 (s, 1H), 8.08 (d, $J$ = 6.6 Hz, 2H), 7.83 – 7.51 (m, 3H), 6.95 (s, 1H), 6.68 (s, 1H) ppm. $^{13}$C NMR (101 MHz, DMSO-d$_6$): δ 182.2, 162.9, 153.7, 149.9, 147.0, 131.8, 131.0, 129.4, 129.1, 126.3, 104.5, 104.3, 94.1 ppm. HPLC purity: 99.0 %

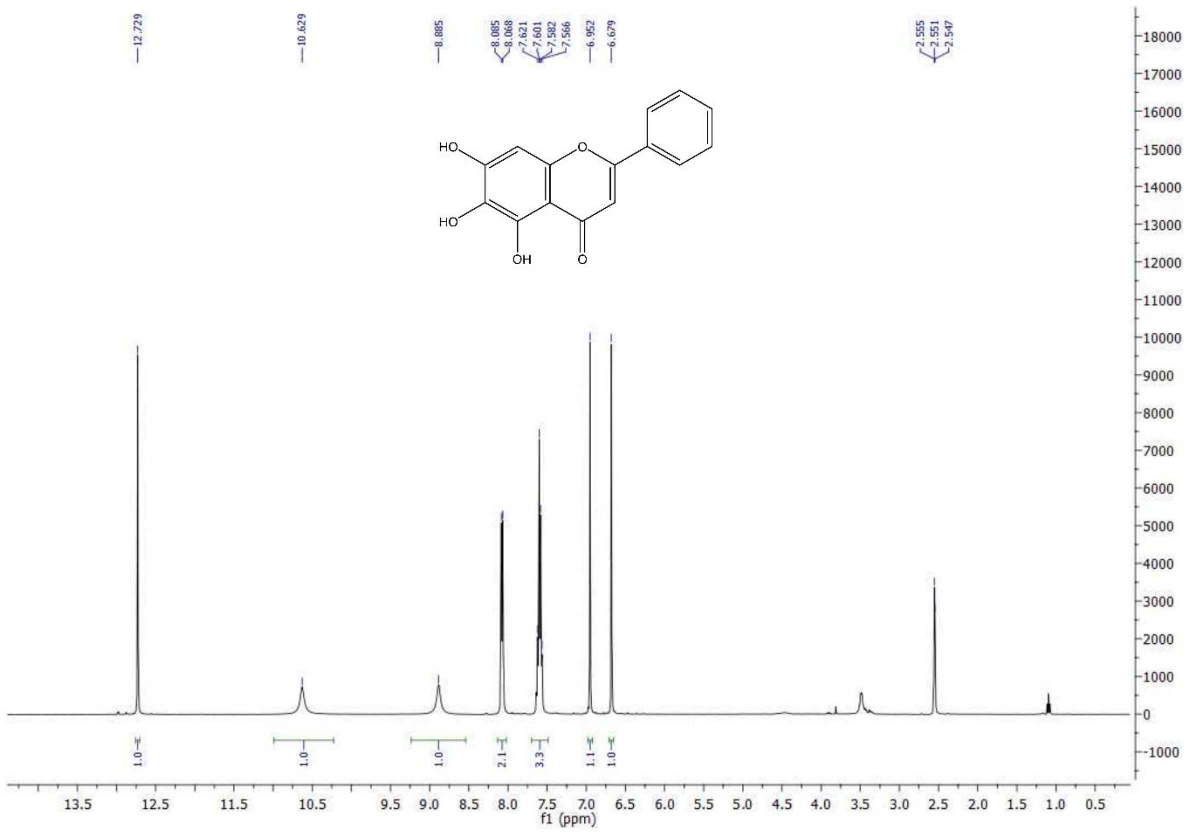


**Figure S20**. $^{1}$H-NMR spectrum of baicalein (**26**)

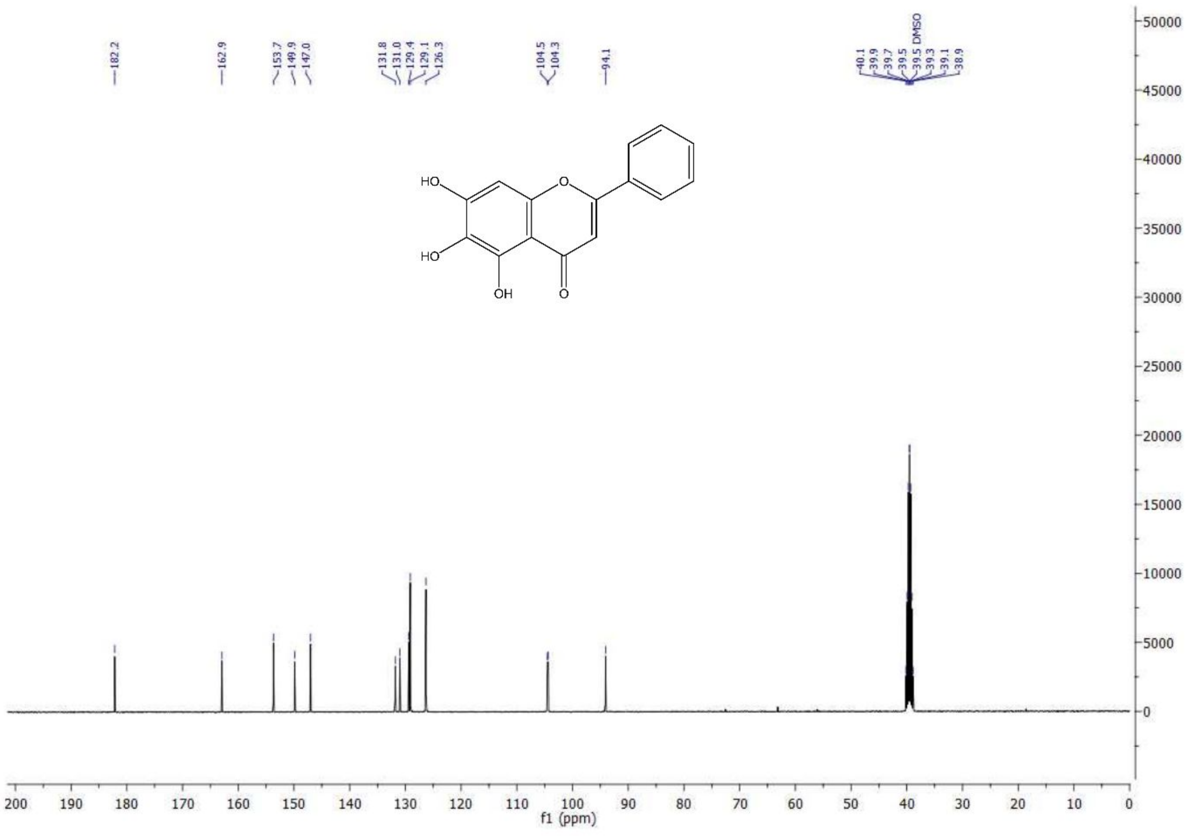


**Figure S21**. $^{13}$C-NMR spectrum of baicalein (**26**)

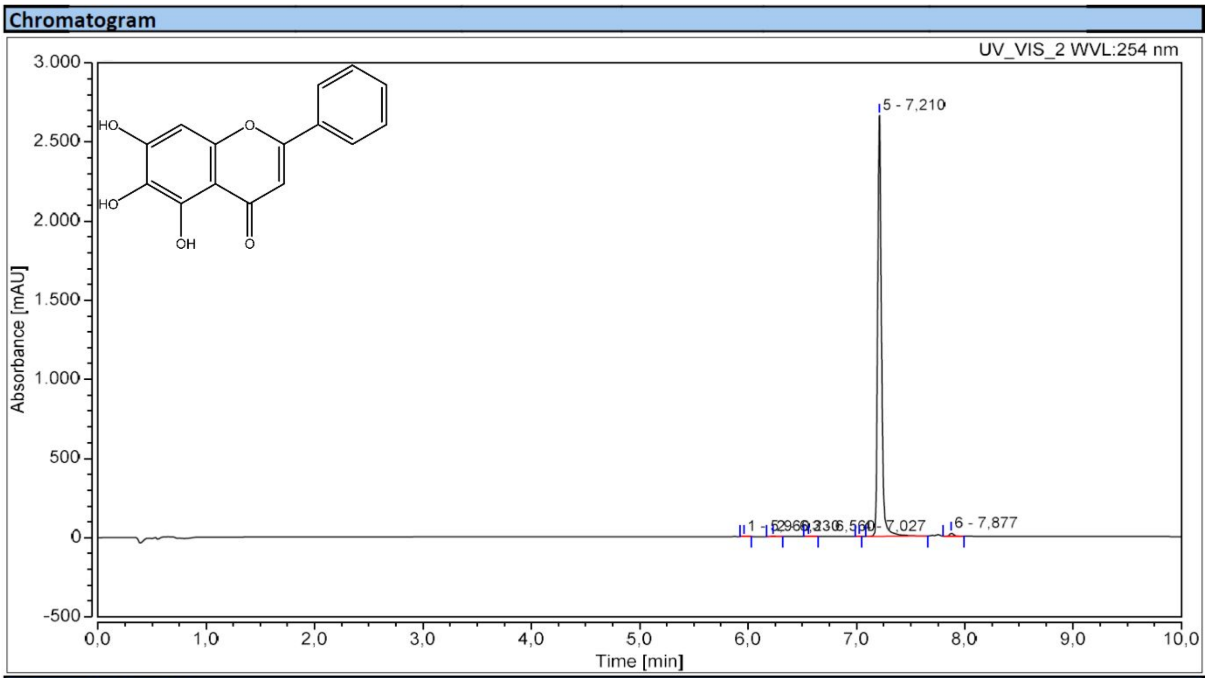


**Integration Results**

| No. | Peak Name | Retention Time min | Area mAU*min | Height mAU | Relative Area % | Relative Height % | Amount n.a. |
|---|---|---|---|---|---|---|---|
| 1 | | 5,960 | 0,075 | 1,653 | 0,07 | 0,06 | n.a. |
| 2 | | 6,230 | 0,160 | 2,305 | 0,14 | 0,09 | n.a. |
| 3 | | 6,560 | 0,040 | 1,126 | 0,04 | 0,04 | n.a. |
| 4 | | 7,027 | 0,025 | 0,758 | 0,02 | 0,03 | n.a. |
| 5 | | 7,210 | 109,643 | 2660,391 | 99,02 | 99,10 | n.a. |
| 6 | | 7,877 | 0,788 | 18,248 | 0,71 | 0,68 | n.a. |
| **Total:** | | | **110,731** | **2684,482** | **100,00** | **100,00** | |

**Figure S22**. HPLC chromatogram of baicalein (**26**)

**Alpinetin** (**48**)

$^{1}$H NMR (400 MHz, DMSO-d$_6$): δ 10.58 (s, 1H), 7.53 – 7.46 (m, 2H), 7.46 – 7.31 (m, 3H), 6.08 (d, *J* = 2.1 Hz, 1H), 6.01 (d, *J* = 2.2 Hz, 1H), 5.49 (dd, *J* = 12.4, 3.0 Hz, 1H), 3.74 (s, 3H), 2.99 (dd, *J* = 16.4, 12.4 Hz, 1H), 2.62 (dd, *J* = 16.4, 3.1 Hz, 1H) ppm. $^{13}$C NMR (101 MHz, DMSO): δ 187.4, 164.4, 164.0, 162.2, 139.2, 128.5, 128.3, 126.4, 104.5, 95.6, 93.3, 78.0, 55.6, 44.9 ppm. HPLC purity: 97.6%

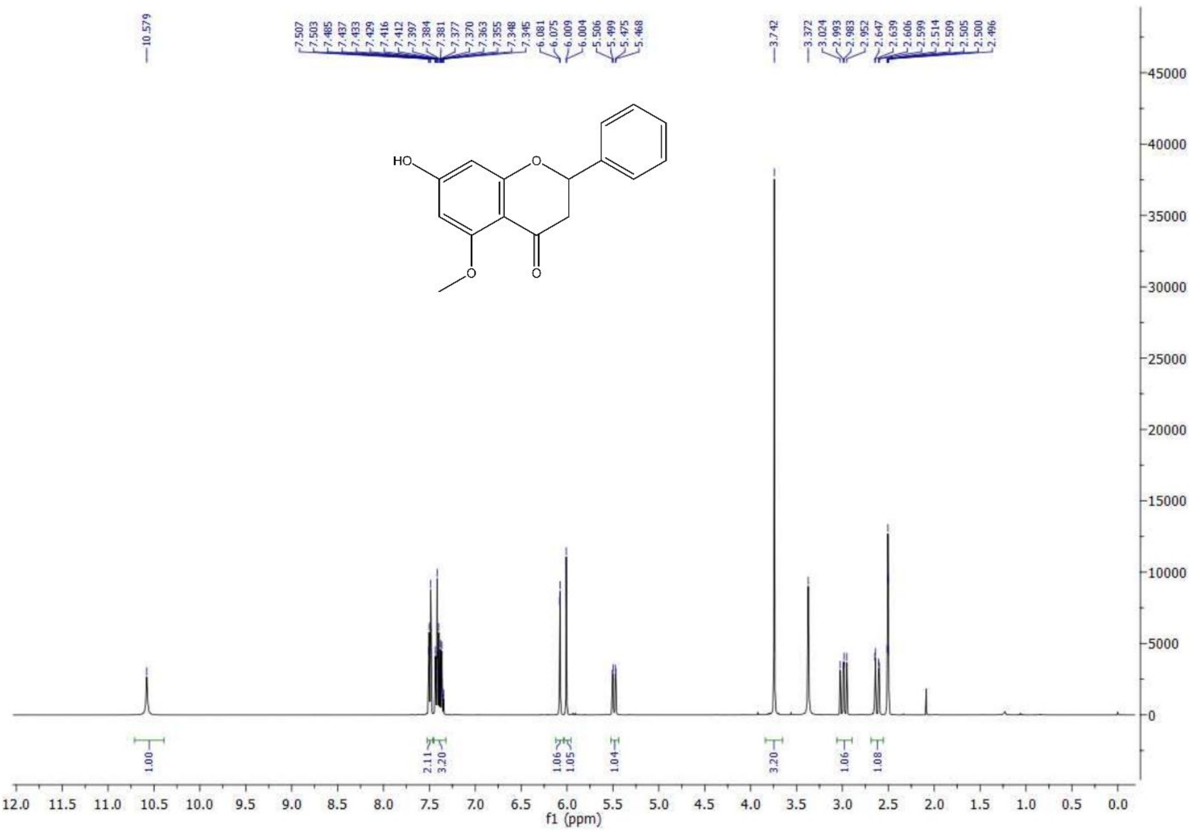

**Figure S23**. $^{1}$H-NMR spectrum of alpinetin (**48**)

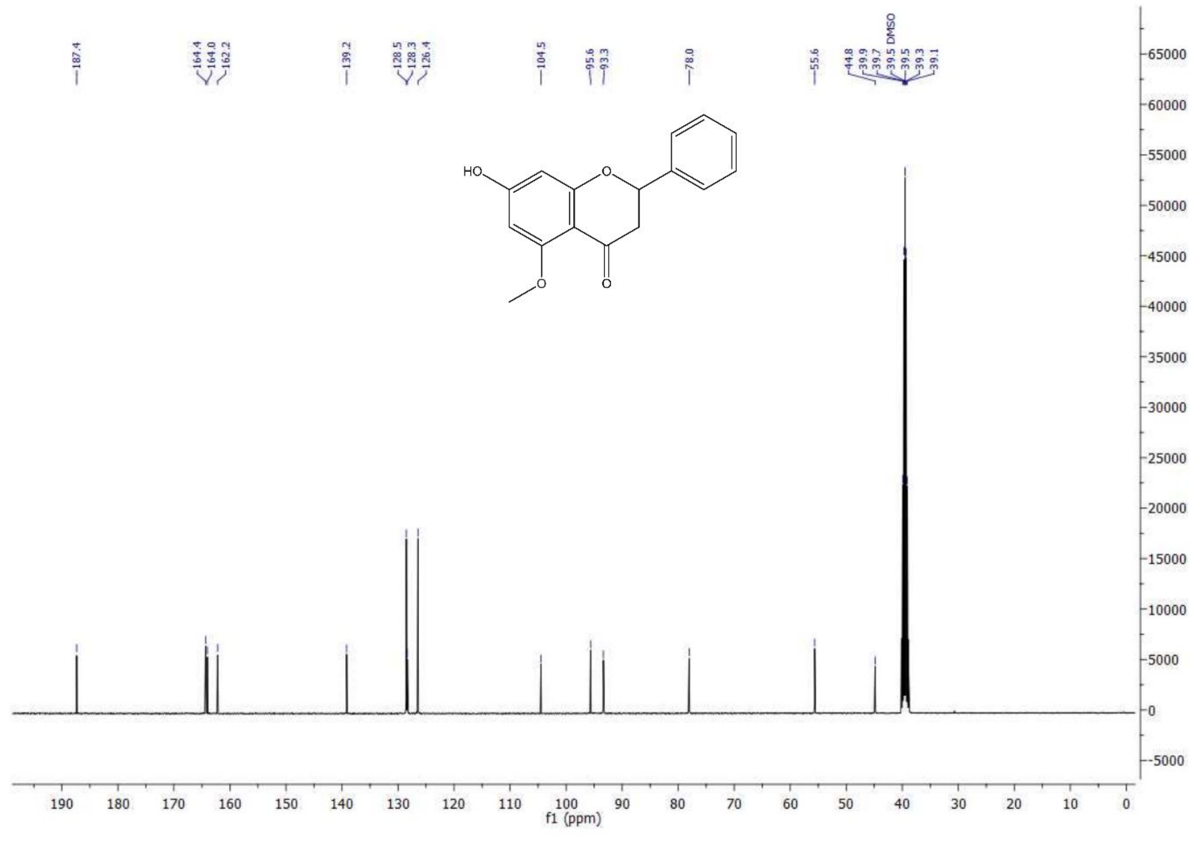

**Figure S24**. $^{13}$C-NMR spectrum of alpinetin (**48**)

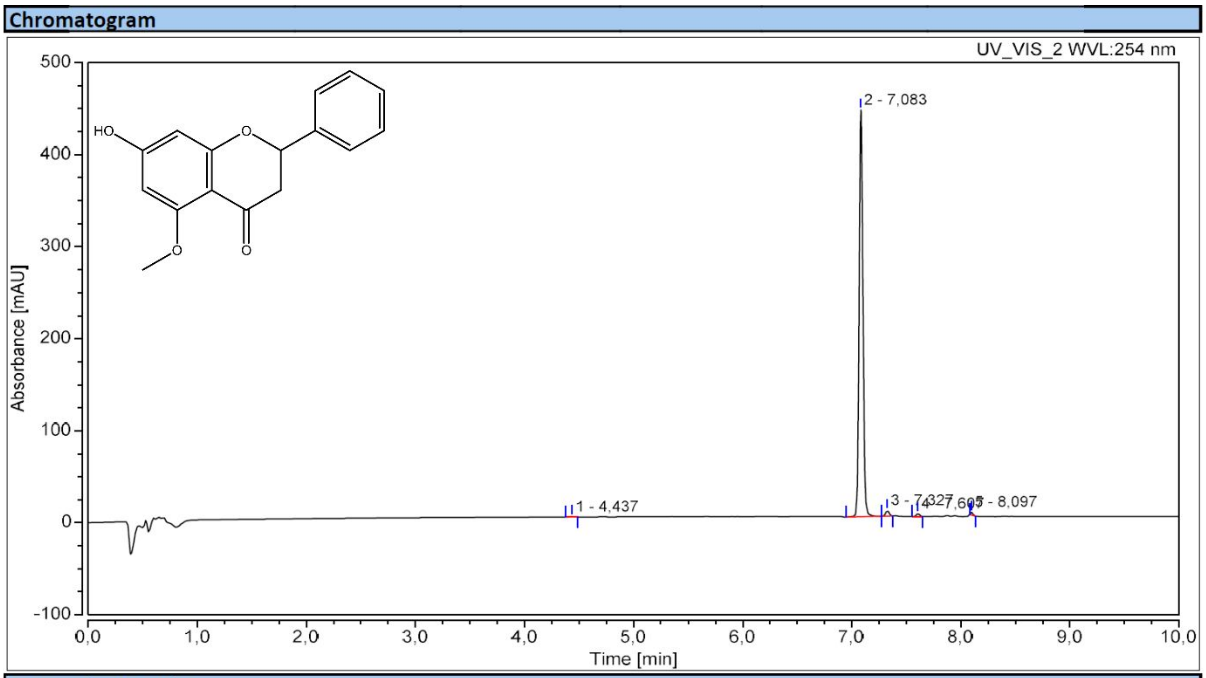


**Integration Results**

| No. | Peak Name | Retention Time min | Area mAU*min | Height mAU | Relative Area % | Relative Height % | Amount n.a. |
|---|---|---|---|---|---|---|---|
| 1 | | 4,437 | 0,023 | 0,433 | 0,13 | 0,10 | n.a. |
| 2 | | 7,083 | 17,708 | 442,172 | 97,61 | 97,27 | n.a. |
| 3 | | 7,327 | 0,222 | 5,974 | 1,22 | 1,31 | n.a. |
| 4 | | 7,607 | 0,107 | 2,964 | 0,59 | 0,65 | n.a. |
| 5 | | 8,097 | 0,082 | 3,045 | 0,45 | 0,67 | n.a. |
| **Total:** | | | **18,142** | **454,588** | **100,00** | **100,00** | |

**Figure S25**. HPLC chromatogram of alpinetin (**48**)

**Ampelopsin** (**49**)

$^1$H NMR (400 MHz, DMSO-d$_6$): δ 11.90 (s, 1H), 10.82 (s, 1H), 8.92 (s, 2H), 8.23 (s, 1H), 6.40 (s, 2H), 5.88 (dd, *J* = 17.2, 2.1 Hz, 2H), 5.77 (d, *J* = 6.2 Hz, 1H), 4.91 (d, *J* = 10.9 Hz, 1H), 4.42 (dd, *J* = 10.9, 6.2 Hz, 1H). $^{13}$C NMR (101 MHz, DMSO-d$_6$): δ 197.7, 166.9, 163.4, 162.6, 145.8, 133.5, 127.2, 107.1, 100.6, 96.1, 95.1, 83.3, 71.7 ppm. HPLC purity: 95.1%

**Figure S26**. $^{1}$H-NMR spectrum of ampelopsin (**49**)

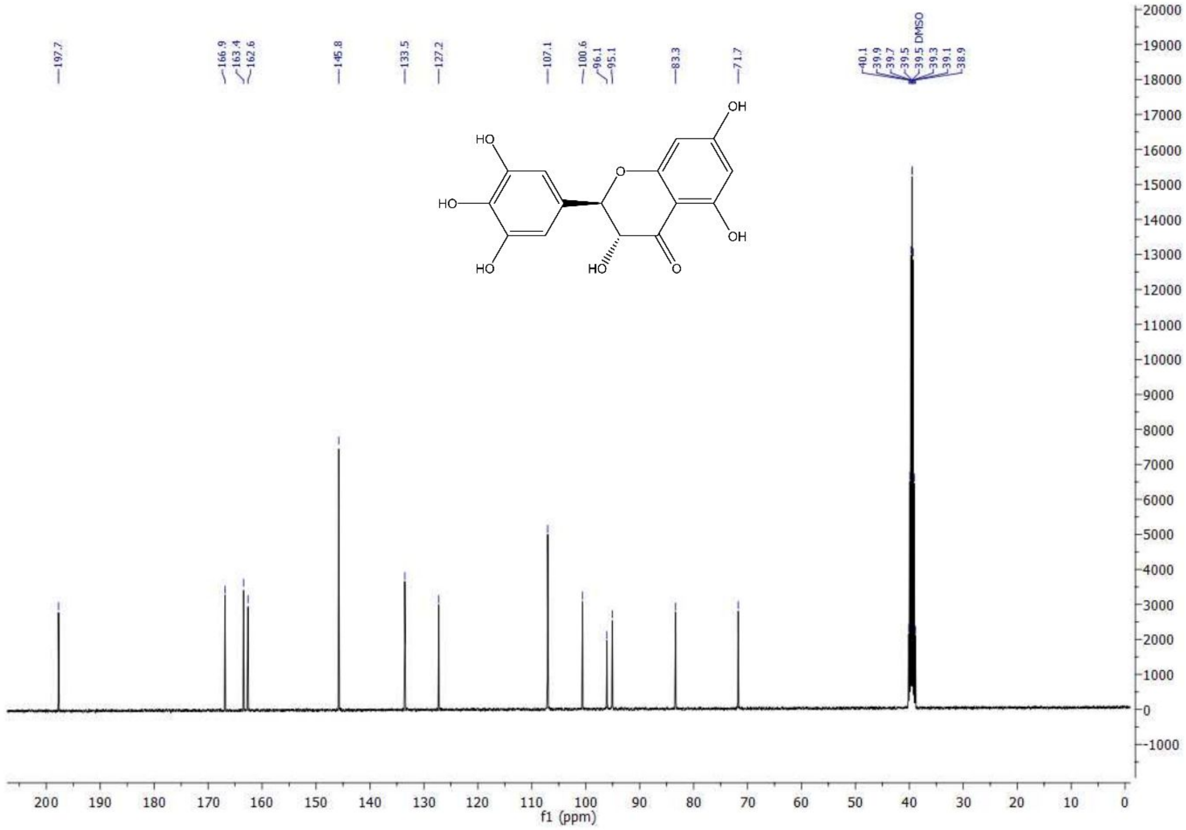


**Figure S27**. $^{13}$C-NMR spectrum of ampelopsin (**49**)

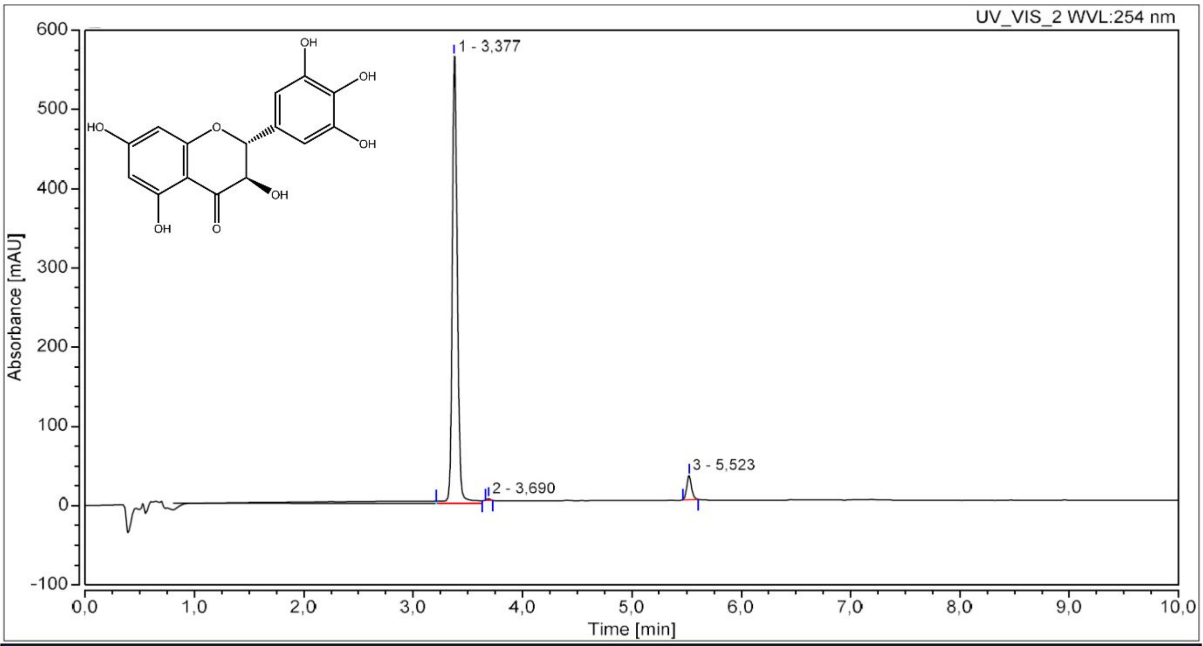


**Integration Results**

| No. | Peak Name | Retention Time<br>min | Area<br>mAU*min | Height<br>mAU | Relative Area<br>% | Relative Height<br>% | Amount<br>n.a. |
|---|---|---|---|---|---|---|---|
| 1 | | 3,377 | 30,913 | 564,448 | 95,08 | 94,59 | n.a. |
| 2 | | 3,690 | 0,066 | 1,847 | 0,20 | 0,31 | n.a. |
| 3 | | 5,523 | 1,533 | 30,461 | 4,72 | 5,10 | n.a. |
| **Total:** | | | **32,512** | **596,756** | **100,00** | **100,00** | |

**Figure S28**. HPLC chromatogram of ampelopsin (**49**)

# Biochemical analysis

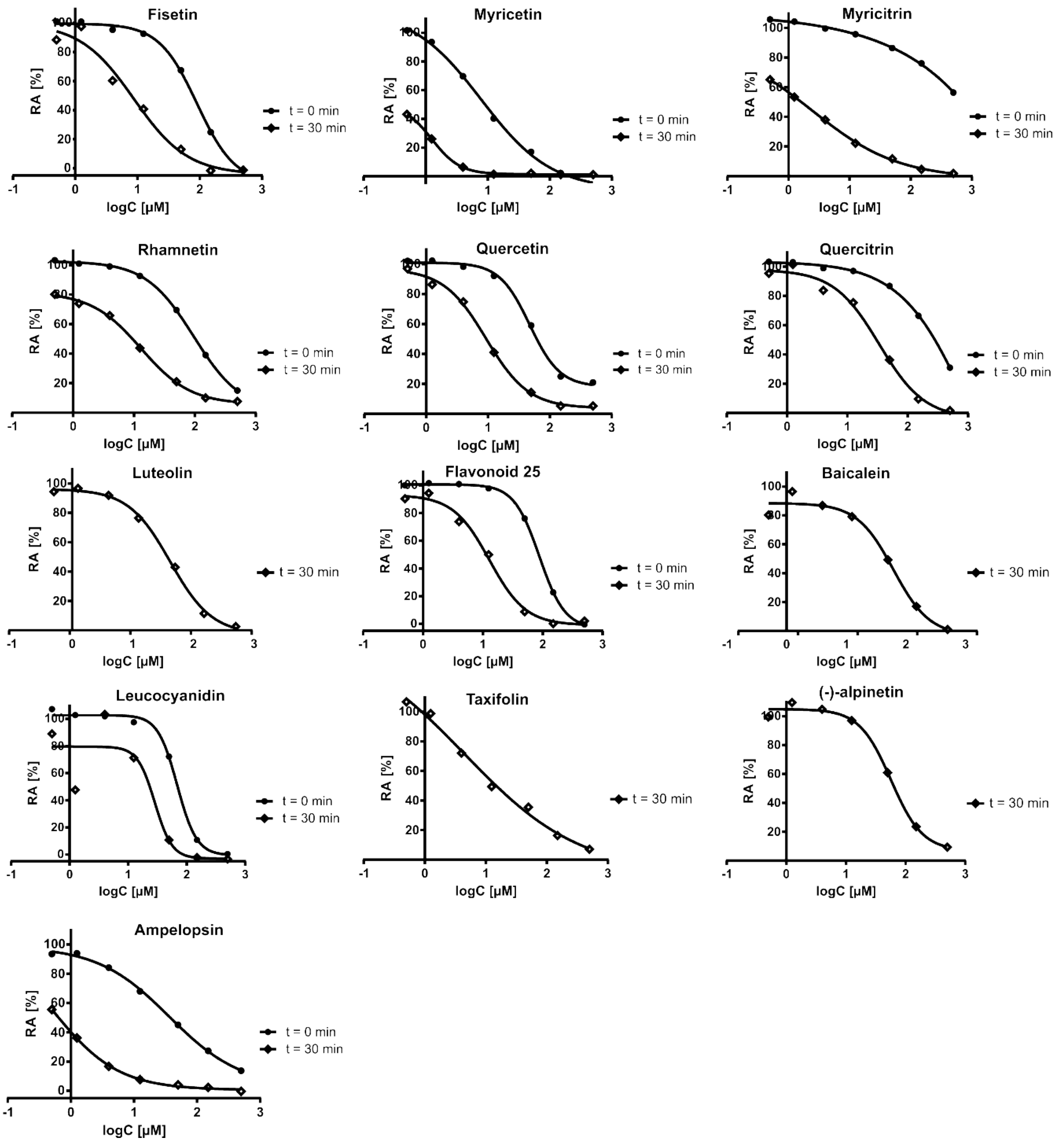


**Figure S29**. Dose-response curves
